# Synthesis of New Optically Active 2-Pyrrolidinones

**DOI:** 10.3390/molecules18010050

**Published:** 2012-12-21

**Authors:** Panagiota Moutevelis-Minakakis, Eleni Papavassilopoulou, Thomas Mavromoustakos

**Affiliations:** Laboratory of Organic Chemistry, Department of Chemistry, University of Athens, Panepistimiopolis, Athens 15771, Greece

**Keywords:** *S*-pyroglutamic acid, 2-pyrrolidinone, imidazole

## Abstract

A new class of optically active 2-pyrrolidinones was synthesized, starting from *S*-pyroglutamic acid, a well known natural chiral synthon. The synthetic design followed led to the insertion of various substituents at positions 1 and 5 of the 2-pyrrolidinone ring, including the imidazole moiety. Some of them possess two or three stereogenic centers, the configuration of which was retained under the mild conditions used. The new compounds also carry an imidazole moiety, which, along with the 2-pyrrolidinone template, may prove pivotal to several biological processes.

## 1. Introduction

Derivatives of 2-pyrrolidinone have shown significant biological and pharmacological activities. Some of them are well known medicines, e.g., 2-oxo-1-pyrrolidine acetamide (piracetam) for patients with seizures, Alzheimer’s and senile dementia, concussion and other neurological problems [1], 1-ethyl-4-(2-morpholin-4-ylethyl)-3,3-diphenyl-pyrrolidin-2-one (doxapram) for patients with respiratory failure [2], *etc.* Moreover, the 2-pyrrolidinone template, considered as an essential pharmacophore group, is also aggregation-inhibiting effects [4], or properties related to the treatment of a variety of diseases incorporated in more complicated chemical structures with anticonvulsant activity [3], pharmacological properties like modulated by H3 receptors, including those associated with the central nervous system [5] and many others.

**Figure 1:**
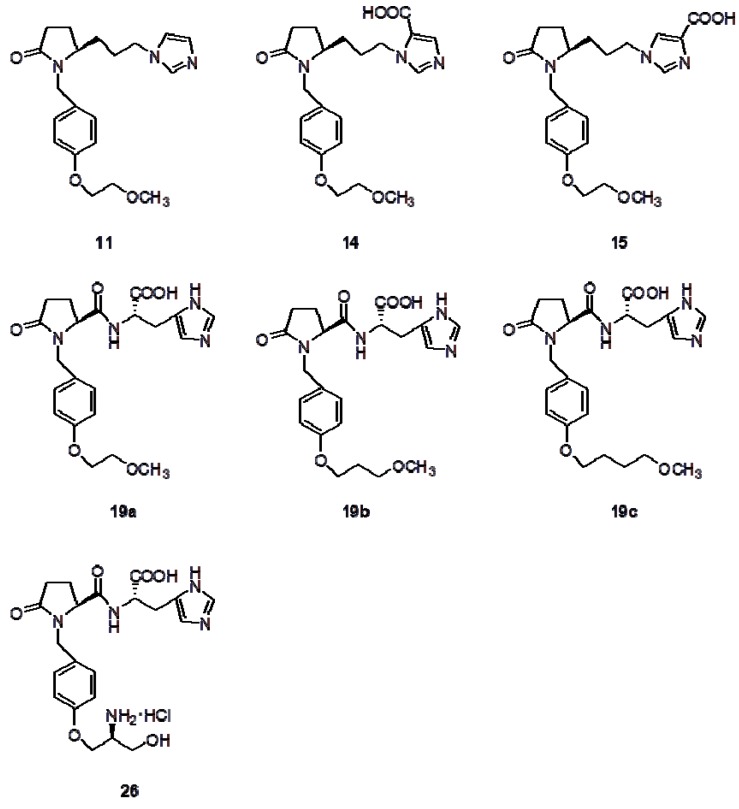
The structure of the new synthesized 2-pyrrolidinones.

In recent years, we have designed and synthesized 2-pyrrolidinones, starting from the naturally derived *S*-pyroglutamic acid (2-oxotetrahydropyrrol-5*S*-carboxylic acid), which is considered as a unique chiral synthon. The asymmetric use of *S*-pyroglutamic acid is based on its two differentiated carbonyls, the properties of which allow an extended derivatization on the 5-membered ring of the starting compound leading to a plethora of natural products, e.g., (−) domoic acid [6], the neurotoxin anatoxin-a [7], biologically interesting compounds, e.g., an inhibitor of angiotensin-converting enzyme (ACE) [8] for the treatment of hypertension as well as to chemical auxiliaries in asymmetric synthesis [9]. The properties and applications of pyroglutamic acid as a versatile building block in asymmetric synthesis has extensively been reviewed in the literature [10,11,12]. As it was mentioned above, in recent years, we have synthesized optically active pyrrolidinones based on the *S*-pyroglutamic acid, in which an imidazole ring has also been inserted. Some of them exhibited antihypertensive and anti-inflammatory activity [13,14,15,16]. In this paper we present the synthesis of seven new compounds (Figure 1), containing up to three stereogenic centers, with predetermined absolute configuration derived from the natural amino acid *S*-pyroglutamic acid, *S*-histidine and *S*-serine. 

## 2. Results and Discussion

The 2-pyrrolidinones shown in Figure 1 possess an *N*-benzyl- type substituent, the 4-position of which is substituted by a methoxyalkyloxy group. In the case of compound **26** the alkoxy substituent is a chiral α-amino alcohol moiety.

**Scheme 1:**
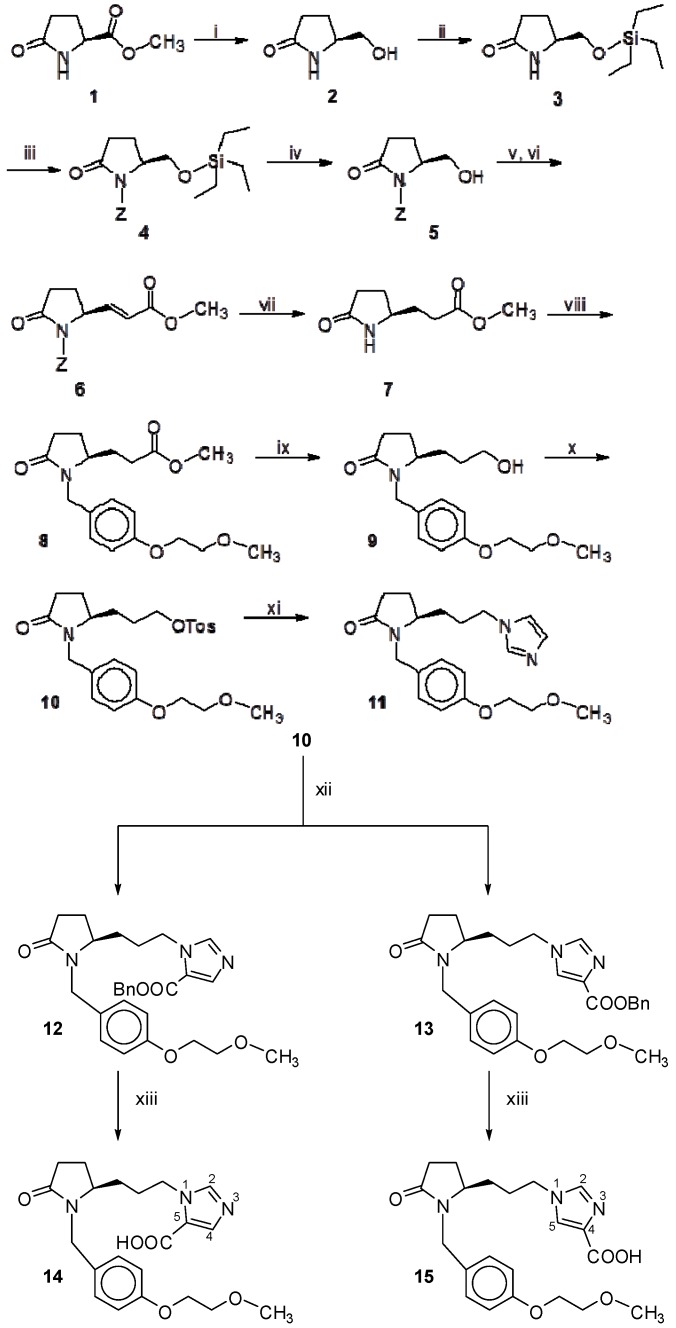
Synthesis of products **11**, **14**, **15**.

**Scheme 2:**
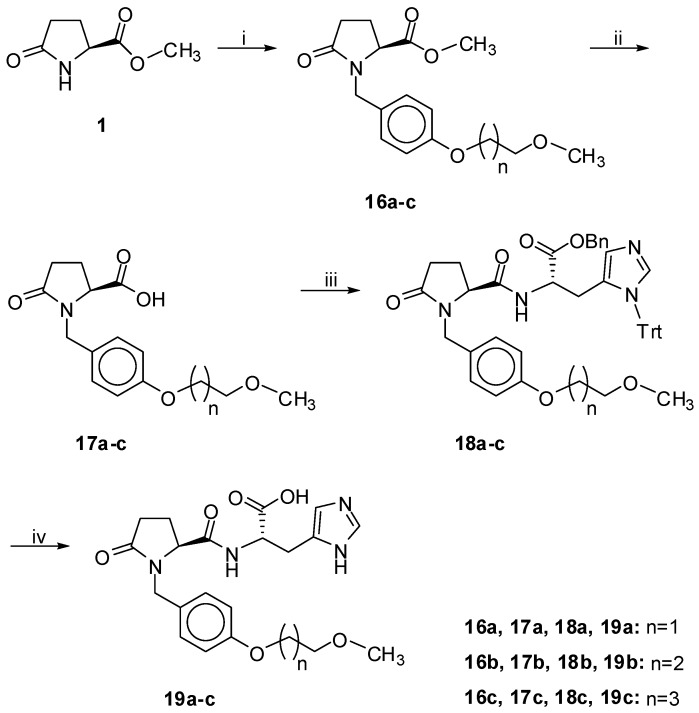
Synthesis of products **19a**–**c**.

**Scheme 3:**
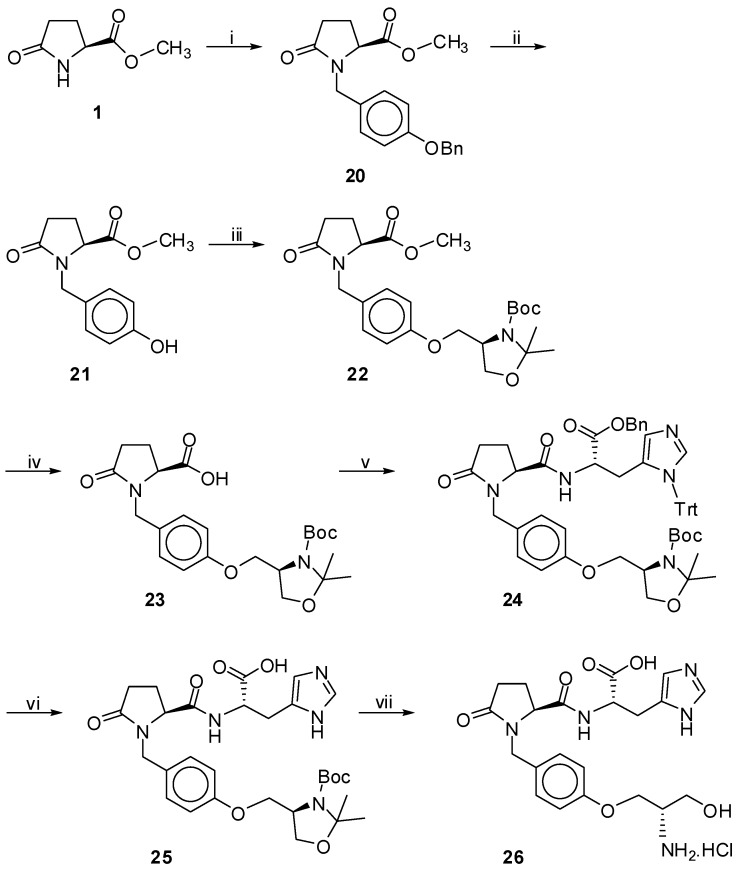
Synthesis of product **26**.

The 5-position of the 2-pyrrolidinone template has retained the absolute configuration of the initial *S*-pyroglutamic acid, regardless of whether the carbon chain is elongated or not. At the other end of this chain, an imidazole ring (with or without substitution) has been inserted (compounds **11**, **14**, **15**). In the case of compounds **19a**–**c** and **26**, the carboxyl group of the starting acid has been coupled to the amino acid *S*-histidine. Our approach for the synthesis of these 2-pyrrolidinones is depicted in Scheme 1, Scheme 2 and Scheme 3. 

**Scheme 4:**
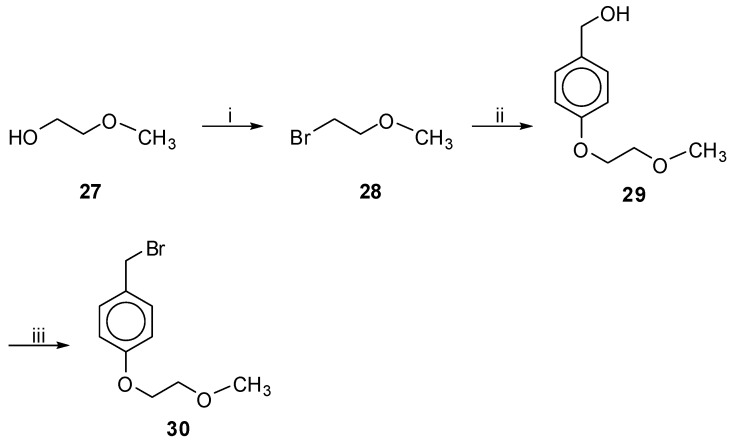
Synthesis of bromide **30**.

As shown in Scheme 1, the *S*-pyroglutaminol (**2**), derived from methyl *S*-pyroglutamate (**1**) using NaBH_4_ in absolute alcohol [17], was protected as the TES ether using TESCl, Et_3_N and DMAP [18] to yield compound **3**. *N*-benzyloxy-carbonylation of **3** with benzyloxycarbonylchloride using NaH in dry THF [19], afforded compound **4**. After deprotection of the TES group using TFA in CH_2_Cl_2_ [20], the resulting alcohol **5** was subjected to Moffatt oxidation [21] yielding the corresponding aldehyde, using DMSO, water soluble carbodiimide (EDC.HCl), pyridine and a trace of TFA. The aldehyde, without being isolated was immediately reacted with the stabilized ylide, phosphoranylidene methyl acetate [22], under the conditions of the Wittig reaction to give alkene **6**. After catalytic hydrogenation, the *N*-unprotected, saturated methylester **7** was obtained, and its NH group was alkylated by benzylbromide **30** (Scheme 4) using NaH in dry DMF [19], to afford compound **8**. The reduction of the ester group of **8** to the corresponding alcohol **9** was accomplished by LiBH_4_ in dry THF [23] and the alcohol **9** was converted to the activated tosyl ester **10**. The desired products **11**–**13** were provided in higher yields (~60%) using imidazole or 1*H*-imidazole-4(5)-carboxylic acid phenylmethyl ester [16] together with Cs_2_CO_3_ in dry DMF at 50 °C. It is known that the nucleophilic strength of nitrogen is enhanced via complexes with Cs^+^, the so called “cesium effect” [24]. After catalytic hydrogenation the final products **14** and **15** were obtained. As shown in Scheme 1, compounds **14** and **15** as well as their precursors **12** and **13** are constitutional isomers in proportion 2:1, depending on which position (4 or 5) on the imidazole ring the substituent is located. In this case the separation of the two isomers was achieved by column chromatography, but we had to identify which isomer corresponds to each isolated compound. In a previous work on similar constitutional isomers [16], we had used 2D NOESY NMR spectroscopy, in order to distinguish whether the carboxyl group is attached on the 4- or 5- carbon of the imidazole ring. According to the ^1^H-NMR spectra of isomers **14** and **15** and taking into account the aforementioned studies, the H-4 of isomer **14** with the carboxylic group at the 5-position of the imidazole ring, resonates at lower field (ca. 7.80 ppm) in comparison to H-5 (ca. 7.68 ppm) of isomer **15** with the carboxylic group at the 4-position. In addition, the diastereotopic proton of the methylene group attached to nitrogen that points towards the carboxylate group in the isomer with substitution at 5-position resonates at lower field (ca. 4.71 ppm) in comparison to the corresponding diastereotopic proton that is not deshielded by the carboxylic group in the isomer with substitution at 4-position and resonates at ca. 4.60 ppm. 

**Scheme 5:**
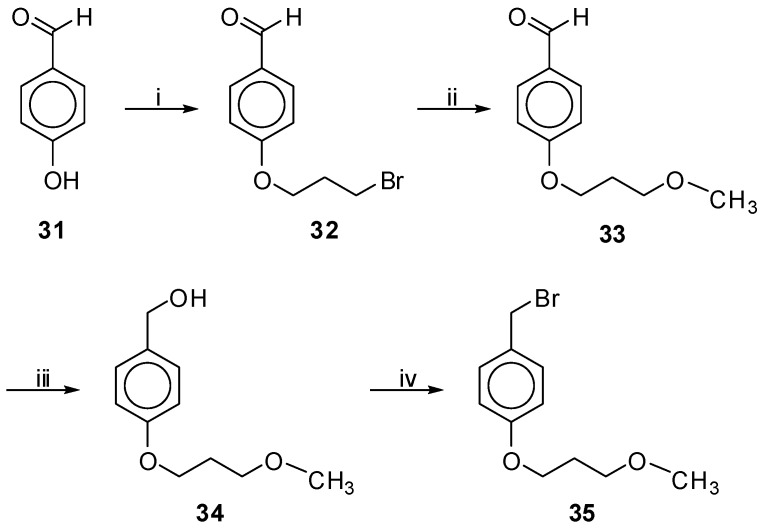
Synthesis of bromide **35**.

**Scheme 6:**
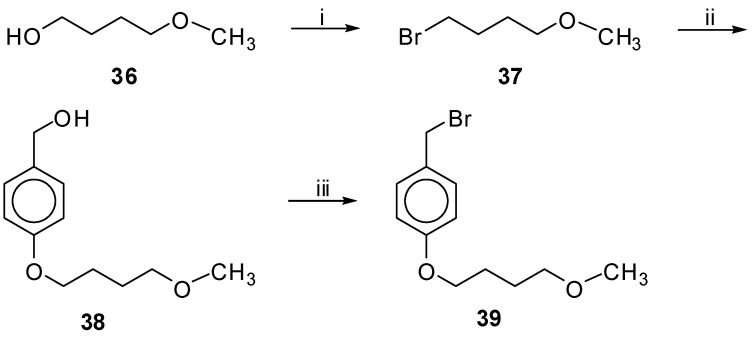
Synthesis of bromide **39**.

**Scheme 7:**
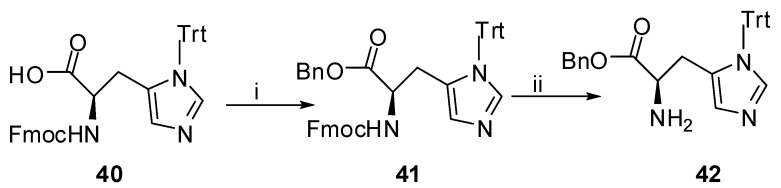
Synthesis of compound **42**.

As shown in Scheme 2, methyl *S*-pyroglutamate was N-benzylated using bromides **30**, **35** and **39 **(Scheme 4, Scheme 5 and Scheme 6). After alkaline hydrolysis of the methyl ester of compounds **16a**–**c**, the resulting carboxylic group was coupled with properly protected *S*-histidine (**42**, Scheme 7) using the water soluble carbodiimide method [25]. The benzylester group and the N-trityl group of the protected amino acid were removed in the end under catalytic hydrogenation. 

Bromides **30**, **35** and **39** were prepared under the conditions depicted in Scheme 4, Scheme 5 and Scheme 6 respectively. Bromides **28** and **37** (Scheme 4 and Scheme 6), prepared from the commercially available alcohols **27** and **36** using PBr_3_ in Et_2_O, respectively [26], were used to convert 4-hydroxybenzyl alcohol to phenol ethers **29** and **38** using K_2_CO_3 _and 18-crown-6 in acetone [27]. Finally, using PBr_3_ in Et_2_O the bromide **30** was obtained in 60% yield, whereas bromide **39** was obtained in 42% yield only by the method of TMS-Cl, NaBr in CH_3_CN [28]. We tried to prepare the desired bromide **39** using many other methods like PBr_3 _in Et_2_O, Tos-Cl/Et_3_N and NaBr in acetone or DMF [29], MeSO_2_Cl/Et_3_N and NaBr in DMF or LiBr in THF [30], without any success. In the case of bromide **35**, we followed a different approach, depicted in Scheme 5, since 3-bromo-propanol was not commercially available. Compound **32** was obtained by the reaction of 4-hydroxy benzaldehyde (**31**) and 1,3-dibromo propane with K_2_CO_3_ in acetone [31]. After conversion of the bromide group of **32** to methyl ether by MeONa [31], the aldehyde **33** was readily subjected to reduction by NaBH_4_ in dry THF to afford alcohol **34**. The relatively low yield of the preparation of compound **32** can be explained by the fact that a parallel Cannizzaro reaction had occurred, under the alkaline conditions of that synthetic step, as revealed from the characterization of the isolated by-products. Both benzaldehydes **32** and **33** were unstable even if they were stored at −4 °C for more than 2–3 h.

As depicted in Scheme 3, a different approach was followed for the synthesis of product **26**. Methyl *S*-pyroglutamate was benzylated at the N-position by 1-(benzyloxy)-4-(bromomethyl) benzene [32] to afford product **20**. After deprotection of the benzylether by catalytic hydrogenation, the phenol group of compound **21** reacted with the cyclic alcohol (*tert*-butyl (4*R*)-4-(hydroxymethyl)-2,2-dimethyl-1,3-oxazolidine-3-carboxylate), a precursor of Garner aldehyde and derived from the methylester of *N*-Boc-*S*-serine [33], under Mitsunobu reaction conditions [34]. The resulting product **22**, was hydrolyzed and coupled with a properly protected derivative of *S*-histidine (**42**, Scheme 7). Catalytic hydrogenation, followed by acidic hydrolysis with 4 N HCl in dioxane, afforded the desired product **26** (Figure 1). Finally the synthesis of H-His(Trt)-OBn (**42**) is depicted in Scheme 7. The commercially available Fmoc-His(Trt)-OH (**40**) was converted to the corresponding benzyl ester (**41)** [35]. The desired, properly protected histidine (**42**) was obtained after Fmoc removal [36]. 

## 3. Experimental

### 3.1. General

All chemicals and solvents were reagent grade and used without purification. Dry THF and extra dry DMF (99.8%) over molecular sieves were purchased from Acros. Melting points were determined on a Büchi 530 apparatus and are uncorrected. Specific rotations were measured at 25 °C on a Perkin–Elmer polarimeter using a 10 cm cell. Nuclear magnetic resonance spectra were obtained on a Varian Mercury spectrometer (^1^H-NMR recorded at 200 MHz and ^13^C-NMR recorded at 50 MHz and they are referenced in ppm relative to TMS as an internal standard). Mass spectra were recorded on a Finnigan Surveyor MSQ Plus with only molecular ions and major peaks being reported with intensities quoted as percentages of the base peak. TLC plates (Silica Gel 60 F254) were purchased from Merck. Visualization of spots was effected with UV light and/or phosphomolybdic acid in EtOH stain. All target compounds possessed 95% purity as determined by combustion analysis. 

### 3.2. General Procedure for the Preparation of Compounds ***28**, **30**, **35**, **37***

To an ice cooled solution of appropriate alcohol **27**, **29**, **34**, **36** (10 mmol) in Et_2_O (25 mL), PBr_3_ (1.41 mL, 15 mmol) was added dropwise under argon. The mixture was stirred at room temperature for 3–7 h and the reaction was quenched by the addition of H_2_O (15 mL) in small portions at 0 °C. The aqueous phase was removed and the organic layer was washed with H_2_O, dried over Na_2_SO_4_ and evaporated *in vacuo*. In the case of products **30** and **35**, purification was achieved by column chromatography using the appropriate solvent systems as it will be defined in each case below. In the case of bromides **28**, **37**, the oily products were not visible either in UV-light or in iodine stain. They were characterized by NMR and used in the next step without further purification. For this reason the yields of their preparation must be considered as crude yields.

*1-Bromo-2-Methoxyethane* (**28**). Prepared from the commercially available alcohol **27**; Yield: 58% (colorless oil). ^1^H-NMR (CDCl_3_): δ 3.69 (t, J = 6.0 Hz, 2H, OCH_2_), 3.45 (t, *J* = 6.0 Hz, 2H, CH_2_Br), 3.38 (s, 3H, CH_3_); ^13^C-NMR: δ 72.3 (OCH_2_), 58.8 (CH_3_), 30.3 (CH_2_Br). Anal. calcd for C_3_H_7_BrO: C, 25.92; H, 5.08; Found: C, 25.68; H, 5.12.

*1-(Bromomethyl)-4-(2-Methoxyethoxy)benzene* (**30**). Prepared from alcohol **18**. Eluent EtOAc–petroleum ether (bp. 40–60 °C), 3:7; white solid; yield 60%; mp. 47–49 °C; Rf 0.50 in EtOAc–petroleum ether (bp. 40–60 °C), 3:7. ^1^H-NMR (CDCl_3_): δ 7.30 (d, *J* = 8.7 Hz, 2H, Ph), 6.87 (d, *J* = 8.7 Hz, 2H, Ph), 4.48 (s, 2H, C*H*_2_Br), 4.09 (t, *J* = 4.5 Hz, 2H, PhOC*H_2_*), 3.73 (t, *J* = 4.8 Hz, 2H, C*H_2_*OCH_3_), 3.43 (s, 3H, C*H*_3_). ^13^C-NMR: δ 158.7, 130.3, 114.7, 70.8 (*C*H_2_OCH_3_), 67.2 (PhO*C*H_2_), 59.1 (*C*H_3_), 33.9 (*C*H_2_Br). Anal. calcd for C_10_H_13_BrO_2_: C, 49.00; H, 5.35; Found: C, 48.95; H, 5.25.

*1-(Bromomethyl)-4-(3-Methoxypropoxy)benzene* (**35**). Prepared from alcohol **34**. Eluent EtOAc–petroleum ether (bp. 40–60 °C), 3:7; yield: 70% (colorless oil); Rf 0.35 in EtOAc–petroleum ether (bp. 40–60 °C), 3:7. ^1^H-NMR (CDCl_3_): δ 7.31 (d, *J* = 8.7 Hz, 2H, Ph), 6.86 (d, *J* = 8.7 Hz, 2H, Ph), 4.50 (s, 2H, C*H*_2_Br), 4.05 (t, *J* = 6.3 Hz, 2H, PhOC*H_2_*), 3.55 (t, *J* = 6.2 Hz, 2H, C*H_2_*ΟCH_3_), 3.35 (s, 3H, C*H*_3_), 2.11–1.98 (m, 2H, CH_2_C*H*_2_CH_2_). ^13^C-NMR: δ 159.1, 130.4, 129.8, 114.7, 69.1 (*C*H_2_OCH_3_), 64.8 (PhO*C*H_2_), 58.7 (*C*H_3_), 34.1 (*C*H_2_Br), 29.5 (CH_2_*C*H_2_CH_2_). Anal. calcd for C_11_H_15_BrO_2_: C, 50.98; H, 5.83; Found: C, 50.82; H, 5.93.

*1-Bromo-4-Methoxybutane* (**37**). Prepared from the commercially available alcohol **36**; Yield: 60% (colorless oil). ^1^H-NMR (CDCl_3_): δ 3.47–3.37 (m, 4H, OC*H_2_*, C*H*_2_Br), 3.33 (s, 3H, C*H_3_*), 2.02–1.88 (m, 2H, C*H*_2_CH_2_Br), 1.78–1.64 (m, 2H, C*H*_2_CH_2_O). ^13^C-NMR: δ 71.7 (O*C*H_2_), 58.6 (*C*H_3_), 33.7 (*C*H_2_Br), 29.6 (*C*H_2_CH_2_Br), 28.2 (*C*H_2_CH_2_O). Anal. calcd for C_5_H_11_BrO: C, 35.95; H, 6.64; Found: C, 35.82; H, 6.59.

*1-(Bromomethyl)-4-(4-Methoxybutoxy)benzene* (**39**). To a stirred solution containing alcohol **38** (35 mg, 0.166 mmol) and NaBr (17 mg, 0.166 mmol) in CH_3_CN (0.5 mL), TMS-Cl (21 μL, 0.166 mmol) was added under argon. After stirring for 1.5h at rt, the solvent was evaporated *in vacuo* and the residue dissolved in Et_2_O was washed with brine and H_2_O. The organic layer was dried over Na_2_SO_4_ and evaporated under reduced pressure. After purification by column chromatography using EtOAc–petroleum ether (bp. 40–60 °C) 1:9 as eluent, the product was obtained as a colorless oil in 42% yield (19 mg). Rf 0.35 in EtOAc-petroleum ether (bp. 40–60 °C) 1: 9. ^1^H-NMR (CDCl_3_): δ 7.27 (d, *J* = 8.6 Hz, 2H, Ph), 6.84 (d, *J* = 8.6 Hz, 2H, Ph), 4.50 (d, *J* = 12.6 Hz, 2H, C*H*_2_Br), 3.95 (t, *J* = 5.9 Hz, 2H, PhOC*H_2_*), 3.42 (t, *J* = 6.2 Hz, 2H, C*H_2_*OCH_3_), 3.33 (s, 3H, C*H*_3_), 1.87–1.68 (m, 4H, CH_2_C*H*_2_C*H*_2_CH_2_). ^13^C-NMR: δ 159.0, 130.3, 114.5, 72.2 (*C*H_2_OCH_3_), 67.5 (PhO*C*H_2_), 58.5 (*C*H_3_), 34.0 (*C*H_2_Br), 26.1, 25.9. Anal. calcd for C_12_H_17_BrO_2_: C, 52.76; H, 6.27; Found: C, 52.82; H, 6.35.

### 3.3. General Procedure for the Preparation of Compounds ***29**, **38***

To a stirred solution of 4-hydroxybenzylalcohol (1.24 g, 10 mmol) in acetone (45 mL), bromides **28** or **37** (10.5 mmol), K_2_CO_3_ (4.14 g, 30 mmol) and 18-crown-6 (0.020 g) were added consecutively. The mixture was refluxed for 3 days. After evaporation of the solvent, the residue was dissolved in EtOAc and washed with brine and H_2_O. The organic layer was dried over Na_2_SO_4_, filtered and evaporated under reduced pressure. Purification was achieved by column chromatography, eluting with the appropriate solvents as defined for each case below.

*[4-(2-Methoxyethoxy)-phenyl]methanol *(**29**). Prepared from bromide **28**. Eluent CHCl_3_–MeOH, 9.5:0.5; Yield: 76% (colorless oil); Rf 0.48 in CHCl_3_–MeOH, 9.5:0.5. ^1^H-NMR (CDCl_3_): δ 7.27 (d, *J* = 8.7 Hz, 2H, Ph), 6.90 (d, *J* = 8.7 Hz, 2H, Ph), 4.61 (s, 2H, C*H*_2_OH), 4.11 (t, *J* = 4.6 Hz, 2H, PhOC*H_2_*), 3.75 (t, *J* = 4.9 Hz, 2H, C*H_2_*OCH_3_), 3.44 (s, 3H, C*H*_3_); ^13^C-NMR: δ 158.2, 133.3, 128.5, 114.5, 70.9 (*C*H_2_OCH_3_), 67.2 (PhO*C*H_2_), 64.8 (*C*H_2_OH), 59.2 (*C*H_3_). Anal. calcd for C_10_H_14_O_3_: C, 65.91; H, 7.74; Found: C, 65.79; H, 7.68.

*[4-(4-Methoxybutoxy) Phenyl]methanol* (**38**). Prepared from bromide **37**. Eluent CHCl_3_–MeOH, 9:1; Yield: 67% (colorless oil); Rf 0.35 in CHCl_3_–MeOH, 9:1. ^1^H-NMR (CDCl_3_): δ 7.26 (d, *J* = 8.7 Hz, 2H, Ph), 6.86 (d, *J* = 8.7 Hz, 2H, Ph), 4.60 (s, 2H, C*H*_2_OH), 3.97 (t, *J* = 6.1 Hz, 2H, PhOC*H_2_*), 3.43 (t, *J* = 6.2 Hz, 2H, C*H_2_*OCH_3_), 3.34 (s, 3H, C*H*_3_), 1.88–1.69 (m, 4H, CH_2_C*H*_2_C*H*_2_CH_2_). ^13^C-NMR: δ 158.6, 132.9, 128.6, 114.5, 72.3 (*C*H_2_OCH_3_), 67.6 (PhO*C*H_2_), 65.0 (*C*H_2_OH), 58.5 (*C*H_3_), 26.2, 26.0. Anal. calcd for C_12_H_18_O_3_: C, 68.54; H, 8.63; Found: C, 68.62; H, 8.59.

*4-(3-Bromopropoxy) Benzaldehyde *(**32**). To a stirred solution of 4-hydroxy-benzaldehyde (1 g, 8.2 mmol) in CH_3_CN (15 mL), K_2_CO_3_ (1.70 g, 12.3 mmol) and 1,3-dibromopropane (8.3 mL, 81.9 mmol) were added consecutively. A tube filled with CaCl_2_ was fixed on the condenser and the mixture was refluxed overnight. After evaporation of the solvent, the residue was dissolved in EtOAc and washed with brine and H_2_O. The organic layer was dried over Na_2_SO_4_, filtered and evaporated under reduced pressure. Purification was achieved by column chromatography using EtOAc–petroleum ether (bp. 40–60 °C) 1:4 as eluent. The product was obtained as colorless oil in 40% yield (0.8 g) and stored at −4 °C, only for a short time. Rf 0.36 in EtOAc–petroleum ether (bp. 40–60 °C) 1:4. ^1^H-NMR (CDCl_3_): δ 9.88 (s, 1H, C*H*O), 7.83 (d, *J* = 8.9 Hz, 2H, Ph), 7.00 (d, *J* = 8.9 Hz, 2H, Ph), 4.19 (t, *J* = 5.8 Hz, 2H, PhOC*H_2_*), 3.60 (t, *J* = 6.3 Hz, 2H, C*H_2_*Br), 2.41–2.29 (m, 2H, CH_2_C*H*_2_CH_2_). ^13^C-NMR: δ 190.7 (*C*HO), 163.6, 131.9, 130.1, 114.7, 65.6 (PhO*C*H_2_), 32.0 (*C*H_2_Br), 29.6 (CH_2_*C*H_2_CH_2_). Anal. calcd for C_10_H_11_BrO_3_: C, 49.41; H, 4.56; Found: C, 49.56; H, 4.39.

*4-(3-Methoxypropoxy)benzaldehyde* (**33**). To a stirred solution of bromide **32** (46 mg, 0.19 mmol) in MeOH (2 mL), a freshly prepared solution of 1N CH_3_ONa/MeOH (200 μL) was added and the mixture was left stirring at rt for 2 days. The reaction was quenched by the addition of H_2_O and the mixture was acidified with aq. 1 N HCl. After evaporation of the solvent, the residue was dissolved in EtOAc and washed with brine and H_2_O. The organic layer was dried over Na_2_SO_4, _filtered and evaporated under reduced pressure. Purification was achieved by column chromatography using EtOAc–petroleum ether (bp. 40–60 °C) 2:3 as eluent. The product was obtained as a colorless oil in 38% yield (14 mg) and was immediately subjected to the following reaction. Rf 0.38 in EtOAc–petroleum ether (bp. 40–60 °C) 2:3. ^1^H-NMR (CDCl_3_): δ 9.87 (s, 1H, C*H*O), 7.82 (d, *J* = 8.8 Hz, 2H, Ph), 7.00 (d, *J* = 8.8 Hz, 2H, Ph), 4.14 (t, *J* = 6.3 Hz, 2H, PhOC*H_2_*), 3.56 (t, *J* = 6.0 Hz, 2H, C*H_2_*Ο), 3.35 (s, 3H, C*H*_3_), 2.14–2.01 (m, 2H, CH_2_C*H*_2_CH_2_). ^13^C-NMR: δ 190.8 (*C*HO), 164.0, 131.9, 129.8, 114.7, 68.8 (*C*H_2_OCH_3_), 65.2 (PhO*C*H_2_), 58.7 (*C*H_3_), 29.4 (CH_2_*C*H_2_CH_2_). MS (ESI): *m/z* = 195 (100) (M+H^+^). Anal. calcd for C_11_H_14_O_3_: C, 68.02; H, 7.27; Found: C, 68.18; H, 7.15.

*[4-(3-Methoxypropoxy) Phenyl]methanol *(**34**). To a stirred solution of aldehyde **33** (63 mg, 0.324 mmol) in dry THF (2 mL) at 0 °C NaBH_4_ (18 mg, 0.487 mmol) was added under argon. The reaction mixture was left stirring at rt overnight. The reaction was quenched by the addition of a 20% solution of AcOH at 0 °C, until the production of the gas was ceased. The organic solvent was evaporated under reduced pressure and the residue was dissolved in EtOAc. The organic phase was washed with 5% aq. NaHCO_3_, brine and H_2_O, dried over Na_2_SO_4_ and evaporated under reduced pressure. After purification with column chromatography using EtOAc–petroleum ether (bp. 40–60 °C) 2:3 as eluent, the product was obtained as a colorless oil in 75% yield (47 mg). Rf 0.25 in EtOAc-petroleum ether (bp. 40–60 °C) 2:3. ^1^H-NMR (CDCl_3_): δ 7.28 (d, *J* = 8.6 Hz, 2H, Ph), 6.89 (d, *J* = 8.6 Hz, 2H, Ph), 4.61 (s, 2H, C*H*_2_OH), 4.05 (t, *J* = 6.3 Hz, 2H, PhOC*H_2_*), 3.55 (t, *J* = 6.2 Hz, 2H, C*H_2_*ΟCH_3_), 3.35 (s, 3H, C*H*_3_), 2.11–1.98 (m, 2H, CH_2_C*H*_2_CH_2_). ^13^C-NMR: δ 158.6, 133.0, 114.5, 69.2 (*C*H_2_OCH_3_), 65.0 (*C*H_2_OH), 64.8 (PhO*C*H_2_), 58.7 (*C*H_3_), 29.5 (CH_2_*C*H_2_CH_2_). MS (ESI): *m/z* = 197 (100) (M+H^+^). Anal. calcd for C_11_H_16_O_3_: C, 67.32; H, 8.22; Found: C, 67.53; H, 8.09.

*(5S)-(Hydroxymethyl)-2-Pyrrolidinone* (**2**). See reference [17].

*5-[[(Triethylsilyl)oxy] Methyl]-2-Pyrrolidinone* (**3**). See reference [18].

### 3.4. General Procedure of for the Preparation of Compounds ***4**, **8**, **16a**–**c**, **20***

To a cooled solution of methyl (S)-pyroglutamate (**1**), *O*-protected-S-pyroglutaminol (**3**), or methylester **7** (1 mmol) in dry THF (5 mL) NaH (60% in paraffin oil, 1.5 mmol) was added in small portions, followed by the addition of the appropriate benzyl bromide (or benzyloxycarbonylchloride in case of compound **4**) (1.1 mmol). The stirring was continued for 15 min at 0 °C, and for 24 h at rt under argon. The reaction was quenched by the addition of a saturated solution of NH_4_Cl at 0 °C, the organic solvent was evaporated and the residue was dissolved in ethyl acetate. The organic layer was washed with brine, dried over Na_2_SO_4_ and evaporated under reduced pressure. The product was purified by column chromatography (Silica Gel 60) using the appropriate solvent systems as defined in each case below.

*Benzyl (5S)-2-oxo-5-{[(triethylsilyl)oxy]methyl}pyrrolidine-1-carboxylate* (**4**). Prepared from compound **3** and benzyloxycarbonyl chloride. Eluent EtOAc–petroleum ether (bp. 40–60 °C), 4:6. The product was obtained as colorless oil in 50% yield. Rf 0.53 in EtOAc–petroleum ether (bp. 40–60 °C), 4:6; [α]_D_ −68.2 (c 1.1, CHCl_3_). ^1^H-NMR (CDCl_3_): δ 7.46–7.32 (m, 5H, Ph), 5.28 (dd, *J_1_* = 12.4 Hz, *J_2_* = 18.4 Hz, 2H, COOC*H*_2_), 4.28–4.20 (m, 1H, NC*H*), 3.88 (dd, *J_1_* = 4.0 Hz, *J_2_* = 10.6 Hz, 1H, C*H*HOSi), 3.67 (dd, *J_1_* = 2.5 Hz, *J_2_* = 10.7 Hz, 1H, C*H*HOSi), 2.84–2.03 (m, 4H, 2xC*H*_2_), 0.88 (t, *J* = 7.6 Hz, 9H, 3 × C*H*_3_), 0.51 (q, *J* = 7.6 Hz, 6H, 3 × (C*H*_2_CH_3_)) ^13^C-NMR: δ 174.7 (*C*ON), 151.3 (*C*OO), 135.2, 128.5, 128.3, 128.2, 67.8 (*C*H_2_OSi), 63.7 (O*C*H_2_Ph), 58.8 (N*C*H), 32.1, 21.2, 6.6 (*C*H_2_CH_3_), 4.1 (CH_2_*C*H_3_). MS (ESI): *m/z* = 364 (100) (M+H^+^). Anal. calcd for C_19_H_29_NO_4_Si: C, 62.78; H, 8.04; N, 3.85 Found: C, 62.83; H, 7.95; N, 3.92.

*Methyl 3-{(2S)-1-[4-(2-Methoxyethoxy)benzyl]-5-oxopyrrolidin-2-yl} propanoate* (**8**). Prepared from compound **7** and bromide **30**. Eluent EtOAc–petroleum ether (bp. 40–60 °C), 9:1. The product was obtained as colorless oil in 40% yield. Rf 0.23 in EtOAc–petroleum ether (bp. 40–60 °C), 1:9; [α]_D_ +5.2 (c 0.92, CHCl_3_). ^1^H-NMR (CDCl_3_): δ 7.13 (d, *J* = 8.6 Hz, 2H, Ph), 6.83 (d, *J* = 8.6 Hz, 2H, Ph), 4.90 (d, *J* = 14.8 Hz, 1H, NCH*H*Ph), 4.09–4.04 (m, 2H, PhOC*H*_2_), 3.85 (d, *J* = 14.8 Hz, 1H, NCH*H*Ph), 3.73–3.68 (m, 2H, C*H*_2_OCH_3_), 3.62 (s, 3H, COOC*H*_3_), 3.42–3.38 (m, 1H, NC*H*), 3.41 (s, 3H, OC*H*_3_), 2.45–1.54 (m, 8H, 4 × C*H*_2_). ^13^C-NMR: δ 174.8 (N*C*O), 173.0 (*C*OO), 158.1, 129.2, 128.7, 114.6, 70.7 (*C*H_2_OCH_3_), 67.1 (PhO*C*H_2_), 59.1 (O*C*H_3_), 55.7 (N*C*H), 51.7 (COO*C*H_3_), 43.3 (N*C*H_2_Ph), 30.0, 29.0, 27.6, 23.3. MS (ESI): *m/z* = 358 (100) (M+Na^+^). Anal. calcd for C_18_H_25_NO_5_: C, 64.46; H, 7.51; N, 4.18 Found: C, 64.51; H, 7.62; N, 4.05.

*Methyl 1-[4-(2-Methoxyethoxy)benzyl]-5-oxo-L-prolinate* (**16a**). Prepared from methyl-*S*-pyroglutamate (**1**) and bromide **30**. Eluent EtOAc. The product was obtained as a colorless oil in 73% yield. Rf 0.42 in EtOAc; [α]_D_ +20.2 (c 1.0, CHCl_3_). ^1^H-NMR (CDCl_3_): δ 7.07 (d, *J* = 8.5 Hz, 2H, Ph), 6.82 (d, *J* = 8.5 Hz, 2H, Ph), 4.87 (d, *J* = 14.7 Hz, 1H, CH*H*Ph), 4.07–4.02 (m, 2H, PhOC*H*_2_), 3.93–3.86 (m, 2H, CH*H*Ph, NC*H*COO), 3.71–3.66 (m, 2H, C*H*_2_OCH_3_), 3.62 (s, 3H, COOC*H*_3_), 3.39 (s, 3H, OC*H*_3_), 2.61–1.92 (m, 4H, 2 × C*H*_2_). ^13^C-NMR: δ 174.8 (N*C*O), 172.1 (*C*OO), 158.2, 129.7, 127.8, 114.6, 70.8 (*C*H_2_OCH_3_), 67.1 (O*C*H_2_), 59.0 (N*C*H), 58.4 (O*C*H_3_), 52.2 (COO*C*H_3_), 44.8 (*C*H_2_Ph), 29.5, 22.6. MS (ESI): *m/z* = 308 (100) (M+Η^+^). Anal. calcd for C_16_H_21_NO_5_: C, 62.53; H, 6.89; N, 4.56 Found: C, 62.58; H, 6.75; N, 4.61.

*Methyl 1-[4-(3-Methoxypropoxy)benzyl]-5-oxo-L-prolinate* (**16b**). Prepared from methyl-*S*-pyroglutamate (**1**) and bromide **35**. Eluent EtOAc. The product was obtained as a colorless oil in 72% yield. Rf 0.44 in EtOAc; [α]_D_ +13.5 (c 1.0, CHCl_3_). ^1^H-NMR (CDCl_3_): δ 7.08 (d, *J* = 8.6 Hz, 2H, Ph), 6.80 (d, *J* = 8.6 Hz, 2H, Ph), 4.90 (d, *J* = 14.6 Hz, 1H, NCH*H*Ph), 3.99 (t, *J* = 6.3 Hz, 2H, PhOC*H*_2_), 3.95–3.90 (m, 1H, NC*H*COO), 3.89 (d, *J* = 4.6 Hz, 1H, NCH*H*Ph), 3.65 (s, 3H, COOC*H*_3_), 3.51 (t, *J* = 6.2Hz, 2H, C*H*_2_OCH_3_), 3.31 (s, 3H, OC*H*_3_), 2.57–1.93 (m, 6H, 3 × C*H*_2_). ^13^C-NMR: δ 174.8 (N*C*O), 172.2 (*C*OO), 158.5, 129.7, 127.5, 114.5, 69.0 (*C*H_2_OCH_3_), 64.7 (PhO*C*H_2_), 58.6 (N*C*H), 58.4 (O*C*H_3_), 44.8 (*C*H_2_Ph), 29.5, 29.4, 22.6. MS (ESI): *m/z* = 322 (100) (M+Η^+^). Anal. calcd for C_17_H_23_NO_5_: C, 63.54; H, 7.21; N, 4.36 Found: C, 63.48; H, 7.28; N, 4.18.

*Methyl 1-[4-(4-Methoxybutoxy)benzyl]-5-oxo-L-prolinate *(**16c**). Prepared from methyl-*S*-pyroglutamate (**1**) and bromide **39**. Eluent EtOAc. The product was obtained as a colorless oil in 66% yield. Rf 0.47 in EtOAc; [α]_D_ +11.7 (c 1.0, CHCl_3_). ^1^H-NMR (CDCl_3_): δ 7.08 (d, *J* = 8.6 Hz, 2H, Ph), 6.79 (d, *J* = 8.6 Hz, 2H, Ph), 4.90 (d, *J* = 14.7 Hz, 1H, NCH*H*Ph), 3.97–3.89 (m, 1H, NC*H*CO), 3.92 (t, *J* = 6.30 Hz, 2H, PhOC*H*_2_), 3.90 (d, *J* = 14.7 Hz, 1H, NCH*H*Ph), 3.65 (s, 3H, COOC*H*_3_), 3.40 (t, *J* = 6.2 Hz, 2H, C*H*_2_OCH_3_), 3.31 (s, 3H, OC*H*_3_), 2.58–1.96 (m, 4H, 2 × C*H*_2_), 1.84-1.67 (m, 4H, (OCH_2_C*H*_2_)_2_). ^13^C-NMR: δ 174.8 (N*C*O), 172.2 (*C*OO), 158.6, 129.7, 127.5, 114.5, 72.2 (*C*H_2_OCH_3_), 67.5 (PhO*C*H_2_), 58.5 (N*C*H), 58.4 (O*C*H_3_), 52.2 (COO*C*H_3_), 44.9 (N*C*H_2_Ph), 29.5, 26.1, 25.9, 22.7. MS (ESI): *m/z* = 336 (100) (M+Η^+^). Anal. calcd for C_18_H_25_NO_5_: C, 64.46; H, 7.51; N, 4.18 Found: C, 64.38; H, 7.48; N, 4.27.

*Methyl 1-[4-(Benzyloxy)benzyl]-5-oxo-L-prolinate* (**20**). Prepared from methyl-*S*-pyroglutamate (**1**) and 1-(benzyloxy)-4-(bromomethyl) benzene [32]. Eluent EtOAc -petroleum ether (bp. 40–60 °C), 7:3. The product was obtained as a colorless oil in 47% yield. Rf 0.35 in EtOAc–petroleum ether (bp. 40–60 °C), 7:3; [α]_D_ +22.5 (c 1, CHCl_3_). ^1^H-NMR (CDCl_3_): δ 7.44–7.28 (m, 5H, Ph), 7.14 (d, *J* = 8.5 Hz, 2H, Ph), 6.92 (d, *J* = 8.5 Hz, 2H, Ph), 5.05 (s, 2H, OC*H*_2_Ph), 4.93 (d, *J* = 14.7 Hz, 1H, NCH*H*Ph), 4.00-3.92 (m, 2H, NCH*H*Ph, NC*H*), 3.66 (s, 3H, C*H*_3_), 2.53–2.04 (m, 4H, 2 × C*H*_2_). ^13^C-NMR: δ 174.8 (N*C*O), 172.1 (*C*OOCH_3_), 158.2, 136.7, 129.7, 128.4, 127.9, 127.8, 127.3, 114.8, 69.8 (O*C*H_2_Ph), 58.5 (N*C*H), 52.2 (*C*H_3_), 44.8 (N*C*HPh), 29.4, 22.6. MS (ESI): *m/z* = 340 (100) (M+Η^+^). Anal. calcd for C_20_H_21_NO_4_: C, 70.78; H, 6.24; N, 4.13 Found: C, 70.62; H, 6.27; N, 4.22.

*Benzyl (2S)-2-(Hydroxymethyl)-5-oxopyrrolidine-1-carboxylate* (**5**). To a stirred solution of compound **4** (1.20 g, 3.30 mmol) in DCM (20 mL), TFA was added (2.02 mL, 26.4 mmol) at room temperature. Once the reaction was finished (45 min), the solvent was evaporated to dryness. The residue was dissolved in toluene and the solvent was evaporated (twice) for the removal of the acid. Finally the residue was dissolved in ethyl acetate and the organic phase was washed with brine to neutral pH. After drying over Na_2_SO_4_ and evaporation of the solvent under reduced pressure, the product was obtained as a pure white solid. Yield: 0.710 g (86%); Rf 0.37 in EtOAc; mp. 83–86 °C; [α]_D_ −54.2 (c 1.0, CHCl_3_). ^1^H-NMR (CDCl_3_): δ 7.37–7.33 (m, 5H, Ph), 5.23 (s, 2H, OC*H*_2_Ph), 4.29–4.23 (m, 1H, NC*H*), 3.95 (dd, *J_1 _*= 3.2 Hz, *J_2_* = 11.8 Hz, 1H, C*H*HOH), 3.66 (dd, *J_1_* = 3.2 Hz, *J_2_* = 11.8 Hz, 1H, C*H*HOH), 3.09 (bs, 1H, OH), 3.86–1.95 (m, 4H, 2 × C*H*_2_). ^13^C-NMR: δ 175.5 (*C*ON), 151.7 (*C*OO), 135.0, 128.6, 128.4, 128.1, 68.0 (O*C*H_2_Ph), 64.1 (*C*H_2_OH), 59.4 (N*C*H), 32.1, 20.9. MS (ESI): *m/z* = 250 (100) (M+Η^+^). Anal. calcd for C_13_H_15_NO_4_: C, 62.64; H, 6.07; N, 5.62 Found: C, 62.59; H, 6.12; N, 5.59.

*Benzyl (2S)-2-[(1E)-3-Methoxy-3-oxoprop-1-en-1-yl]-5-oxopyrrolidine-1-carboxylate* (**6**). Alcohol **5** (0.22 g, 0.882 mmol) was dissolved in a mixture of dry toluene (5 mL) and dry DMSO (2.5 mL), followed by the addition of EDC·HCl (0.51 g, 2.64 mmol), the dropwise addition of dry pyridine (0.25 mL, 3.04 mmol) and finally TFA (33.2 μL, 0.441 mmol) under argon. After stirring at rt for 1.5 h the reaction was quenched by the addition of CHCl_3_ and the solution was washed with brine and H_2_O. The organic layer was dried over Na_2_SO_4_, filtered and evaporated under reduced pressure. The resulting crude aldehyde was subjected to Wittig reaction without further purification. More specifically, it was dissolved in dry THF (6 mL), methyl (triphenylphosphoranylidene)acetate (0.32 g, 0.95 mmol) was added and the mixture was refluxed under argon for 1 h. The reaction was quenched by the addition of saturated solution of NH_4_Cl. The solvent was evaporated under reduced pressure and the residue dissolved in EtOAc was successively washed with NH_4_Cl, H_2_O and brine. The organic layer was dried over Na_2_SO_4, _filtered and evaporated under reduced pressure. After purification by column chromatography using EtOAc as eluent, the product was obtained in 60% yield (0.16 g) as a colorless oil. Rf 0.63 in EtOAc; [α]_D_ −71.1 (c 1.0, CHCl_3_). ^1^H-NMR (CDCl_3_): δ 7.38–7.31 (m, 5H, Ph), 6.89 (dd, *J_1_* = 6.3 Hz, *J_2 _*= 6.2 Hz, 1H, CHC*H*COOCH_3_), 5.25 (dd, *J_1_* = 12.3 Hz, *J_2_* = 12.2 Hz, 2H, C*H*_2_Ph), 4.87–4.81 (m, 1H, NC*H*), 3.73 (s, 3H, OC*H*_3_), 2.64–1.82 (m, 4H, 2 × C*H*_2_). ^13^C-NMR: δ 173.0 (N*C*OCH_2_), 166.0 (*C*OOCH_3_), 150.8 (*C*OOCH_2_), 145.1, 134.8, 128.5, 128.4, 128.2, 121.5 (*C*HCOOCH_3_), 68.2 (O*C*H_2_Ph), 57.8 (N*C*H), 51.7 (*C*H_3_), 30.7, 23.8. MS (ESI): *m/z* = 326 (100) (M+Na^+^). Anal. calcd for C_17_H_17_NO_5_: C, 63.36; H, 5.65; N, 4.62 Found: C, 63.29; H, 5.48; N, 4.75.

*(5R)-5-(3-Hydroxypropyl)-1-[4-(2-methoxyethoxy)benzyl]-pyrrolidin-2-one* (**9**). To a two necked flask, LiBH_4_ (14 mg, 0.656 mmol) was suspended in dry THF (0.5 mL) under argon at rt. A solution of compound **8** (0.110g, 0.656 mmol) in dry THF (1.5 mL) was added dropwise and the reaction mixture was stirred for 10 h. The reaction was quenched by the addition of a 20% aq. solution of AcOH at 0 °C until gas production ceased. The excess of acetic acid was neutralized by the addition of a small quantity of Na_2_CO_3_. The organic solvent was evaporated under reduced pressure and the residue was dissolved in EtOAc. The organic phase was then washed with brine, dried over Na_2_SO_4_ and evaporated under reduced pressure. After purification with column chromatography using EtOAc–MeOH 9:1 as eluent, the product was obtained as a colorless oil in 87% yield (87 mg). Rf 0.34 in EtOAc–MeOH 9:1; [α]_D_ +7.6 (c 0.95, CHCl_3_). ^1^H-NMR (CDCl_3_): δ 7.13 (d, *J* = 8.6 Hz, 2H, Ph), 6.84 (d, *J* = 8.6 Hz, 2H, Ph), 4.89 (d, *J* = 14.8 Hz, 1H, NCH*H*Ph), 4.10–4.05 (m, 2H, PhOC*H*_2_), 3.88 (d, *J* = 14.8 Hz, 1H, NCH*H*Ph), 3.74–3.70 (m, 2H, C*H*_2_OCH_3_), 3.64–3.54 (m, 2H, C*H*_2_OH), 3.42 (s, 4H, NC*H*, C*H*_3_), 2.47–1.36 (m, 8H, 4 × C*H*_2_). ^13^C-NMR: δ 175.1 (N*C*O), 158.1, 129.2, 128.8, 114.6, 70.9 (*C*H_2_OCH_3_), 67.1 (PhO*C*H_2_), 62.2 (*C*H_2_OH), 59.2 (*C*H_3_), 56.6 (N*C*H), 43.4 (N*C*H_2_Ph), 30.2, 28.9, 27.3, 23.7. MS (ESI): *m/z* = 308 (90) (M+H^+^).Anal. calcd for C_17_H_25_NO_4_: C, 66.43; H, 8.20; N, 4.56 Found: C, 66.38; H, 8.26; N, 4.42.

*3-{(2R)-1-[4-(2-Methoxyethoxy)benzyl]-5-oxopyrrolidin-2-yl}propyl 4-Methylbenzenesulfonate* (**10**). To an ice cooled stirred solution of alcohol **9** (35 mg 0.114 mmol) in DCM (2 mL), p-toluenesulfonylchloride (43 mg, 0.228 mmol) was added, followed by the addition of Et_3_N (17.5 μL). The reaction mixture was stirred at 0 °C for 10 min and overnight at room temperature. The organic layer was subsequently washed with a 1N HCl aq. solution, brine, 5% aq. solution of NaHCO_3_ and brine. After drying over Na_2_SO_4_ and evaporation in vacuo, the residue was purified by column chromatography using EtOAc as eluent, and the product was obtained as a yellowish oil in 72% yield (37 mg). Rf 0.35 in EtOAc; [α]_D_ +5.6 (c 1, CHCl_3_). ^1^H-NMR (CDCl_3_): δ 7.75 (d, *J* = 8.3 Hz, 2H, Ph), 7.34 (d, *J* = 8.0 Hz, 2H, Ph), 7.10 (d, *J* = 8.6 Hz, 2H, Ph), 6.85 (d, *J* = 8.6 Hz, 2H, Ph), 4.83 (d, *J* = 14.9 Hz, 1H, NCH*H*Ph), 4.11–4.06 (m, 2H, PhOC*H*_2_), 3.95 (t, *J* = 5.9 Hz, 2H, C*H*_2_OSO_2_), 3.83 (d, *J* = 14.9Hz, 1H, NCH*H*Ph), 3.75–3.71 (m, 2H, C*H*_2_OCH_3_), 3.43 (s, 3H, OC*H*_3_), 3.39-3.31 (m, 1H, NC*H*), 2.44 (s, 3H, C*H*_3_), 2.41–1.50 (m, 8H, 4 × C*H*_2_). ^13^C-NMR: δ 174.9 (N*C*O), 158.1, 144.9, 132.8, 129.9, 129.2, 128.7, 127.8, 114.7, 70.9 (*C*H_2_OCH_3_), 69.9 (*C*H_2_OSO_2_), 67.2 (PhO*C*H_2_), 59.2 (*C*H_3_), 56.1 (N*C*H), 43.5 (N*C*H_2_Ph), 30.1, 28.7, 23.9, 23.5, 21.6 (Ph*C*H_3_). MS (ESI): *m/z* = 462 (100) (M+H^+^). Anal. calcd for C_24_H_31_NO_6_S: C, 62.45; H, 6.77; N, 3.03 Found: C, 62.32; H, 6.82; N, 3.18.

### 3.5. General Procedure of for the Preparation of Compounds ***11**–**13***

Imidazole or 1*H*-imidazole-4(5)-carboxylic acid phenylmethyl ester [16], (1.2 mmol) and cesium carbonate (0.39 g, 1.2 mmol) were dissolved in dry DMF (3 mL) under argon. After stirring of the mixture for 30 min at 50 °C, a solution of tosyl ester **10** (1 mmol) dissolved in dry DMF (3 mL) was added and the stirring was continued overnight at 50 °C under argon. After evaporation of DMF in high vacuo, the residue was dissolved in EtOAc and the organic phase was washed with brine to neutral pH, dried over Na_2_SO_4_ and evaporated under reduced pressure. The residual product was purified by column chromatography (silica gel) using the appropriate solvent systems as it will be defined, in each case, below.

*(5R)-5-[3-(1H-Imidazol-1-yl)propyl]-1-[4-(2-methoxyethoxy)benzyl]-pyrrolidin-2-one* (**11**). Prepared from tosyl ester** 10** and imidazole as a colorless oil. Eluent EtOAc–MeOH, 7:3. Yield: 58%. Rf 0.29 in EtOAc–MeOH, 7:3. [α]_D_ +4.1 (c 0.8, MeOH). ^1^H-NMR (CD_3_OD): δ 7.61 (s, 1H, NC*H*N), 7.11 (d, *J* = 8.6 Hz, 2H, Ph), 7.07 (s, 1H, CH_2_NC*H*CHN), 6.98 (s, 1H, CH = NC*H* = CHN), 6.89 (d, *J* = 8.6 Hz, 2H, Ph), 4.69 (d, *J* = 14.9 Hz, 1H, NCH*H*Ph), 4.12–4.07 (m, 2H, PhOC*H*_2_), 4.03–3.93 (m, 3H, NCH*H*Ph, C*H*_2_N), 3.75–3.71 (m, 2H, C*H*_2_OCH_3_), 3.52–3.42 (m, 1H, NC*H*), 3.41 (s, 3H, C*H*_3_), 2.47–1.30 (m, 8H, 4 × C*H*_2_). ^13^C-NMR: δ 177.8 (N*C*O), 159.8, 138.5, 130.4, 130.1, 129.1, 120.6, 115.8, 72.2 (*C*H_2_OCH_3_), 68.5 (PhO*C*H_2_), 59.3 (*C*H_3_), 58.7 (CON*C*H), 47.7 (CH_2_N), 44.7 (N*C*H_2_Ph), 31.2, 30.6, 26.9, 24.5. MS (ESI): *m/z* = 358 (100) (M+H^+^). Anal. calcd for C_20_H_27_N_3_O_3_: C, 67.20; H, 7.61; N, 11.76 Found: C, 67.28; H, 7.52; N, 11.82.

*Benzyl 1-(3-{(2R)-1-[4-(2-Methoxyethoxy)benzyl]-5-oxopyrrolidin-2-ylpropyl)-1H-imidazole-5-carboxylate *(**12**). Prepared from tosyl ester **10** and 1*H*-imidazole-4(5)-carboxylic acid phenylmethyl ester [16], as a mixture with its constitutional isomer **13**. Eluent EtOAc-MeOH, 9:1. Yield: 55% (colorless oil). Rf 0.42 in EtOAc–MeOH, 7:3. [α]_D_ +2.8 (c 1, CHCl_3_). ^1^H-NMR (CDCl_3_): δ 7.78 (s, 1H, NC*H*N), 7.58 (s, 1H, NC*H*COO), 7.40–7.34 (m, 5H, Ph), 7.08 (d, *J* = 8.6 Hz, 2H, Ph), 6.85 (d, *J* = 8.6 Hz, 2H, Ph), 5.28 (s, 2H, OC*H*_2_Ph), 4.78 (d, *J* = 14.9 Hz, 1H, NCH*H*Ph), 4.20 (t, *J* = 6.7 Hz, 2H, C*H*_2_N), 4.10–4.06 (m, 2H, PhO*C*H_2_), 3.87 (d, *J* = 14.9 Hz, 1H, NCH*H*Ph), 3.74–3.70 (m, 2H, C*H*_2_OCH_3_), 3.42 (s, 3H, C*H*_3_), 3.41–3.33 (m, 1H, NC*H*), 2.43–1.24 (m, 8H, 4 × C*H*_2_). ^13^C-NMR: δ 174.9 (N*C*O), 159.8 (COO), 158.1, 141.8, 137.9, 135.5, 129.1, 128.8, 128.6, 128.5, 128.3, 128.0, 114.7, 70.9 (*C*H_2_OCH_3_), 67.2 (PhO*C*H_2_), 66.2 (COO*C*H_2_Ph), 59.1 (*C*H_3_), 56.3 (N*C*H), 46.6 (*C*H_2_NCCOO), 43.6 (N*C*H_2_Ph), 30.1, 29.6, 26.0, 23.6. MS (ESI): *m/z* = 492 (100) (M+H^+^). Anal. calcd for C_28_H_33_N_3_O_5_: C, 68.41; H, 6.77; N, 8.55 Found: C, 68.32; H, 6.60; N, 8.57.

*Benzyl 1-(3-{(2R)-1-[4-(2-Methoxyethoxy)benzyl]-5-oxopyrrolidin-2-yl}propyl)-1H-imidazole-4-carboxylate* (**13**). Prepared from tosyl ester **10** and 1*H*-imidazole-4(5)-carboxylic acid, phenylmethyl ester [16], as a mixture with its constitutional isomer **12**. After the separation of isomer **12**, the elution system (EtOAc–MeOH, 9:1) was gradually changed to the more polar EtOAc–MeOH, 3:2 in order to recover isomer 13. Yield: 30% (colorless oil). Rf 0.56 in EtOAc–MeOH, 3:2. [α]_D_ +9.9 (c 1, CHCl_3_). ^1^H-NMR (CDCl_3_): δ 7.53 (s, 1H, NC*H*N), 7.51 (s, 1H, NC*H*COO), 7.46–7.31 (m, 5H, Ph), 7.07 (d, *J* = 8.7 Hz, 2H, Ph), 6.84 (d, *J* = 8.7 Hz, 2H, Ph), 6.34 (s, 2H, COOC*H*_2_Ph), 4.73 (d, *J* = 14.9 Hz, 1H, NCH*H*Ph), 4.09–4.05 (m, 2H, PhO*C*H_2_), 3.97–3.82 (m, 3H, NCH*H*Ph, C*H*_2_N), 3.74–3.69 (m, 2H, C*H*_2_OCH_3_), 3.43 (s, 3H, C*H*_3_), 3.41–3.36 (m, 1H, NC*H*), 2.45–1.24 (m, 8H, 4 × C*H*_2_). ^13^C-NMR: δ 174.9 (N*C*O), 162.2 (COO), 158.2, 137.9, 136.0, 133.6, 129.1, 128.7, 128.5, 128.4, 128.2, 124.9, 114.7, 70.9 (*C*H_2_OCH_3_), 67.2 (PhO*C*H_2_), 66.2 (COO*C*H_2_Ph), 59.2 (*C*H_3_), 56.4 (N*C*H), 47.3 (*C*H_2_NCHN), 43.9 (N*C*H_2_Ph), 30.1, 29.7, 25.8, 23.6. MS (ESI): *m/z* = 492 (100) (M+H^+^). Anal. calcd for C_28_H_33_N_3_O_5_: C, 68.41; H, 6.77; N, 8.55 Found: C, 68.38; H, 6.82; N, 8.45.

### 3.6. General Procedure for the Preparation of Compounds ***7**, **14**, **15**, **19a**–**c**, **21**, **25***

A mixture of a compound possessing a double bond and also the *N*- benzyloxycarbonyl group (compound **6**), benzyl ester (compounds **12**,**13**), benzyl ether (compound **20**) or benzyl ester and* N*-trityl imidazole group (compounds **18a**–**c**, **24**) (1 mmol) in MeOH (10 mL) and 10% palladium on activated carbon (0.02 g or 0.04 g for compounds **6**, **18a**–**c**, **24**) was hydrogenated for 1.5–3 h (overnight for compound **6**) under atmospheric conditions. After filtration through a pad of Celite, the solvent was removed *in vacuo* to give the deprotected final compound in almost quantitative yield and high purity. In the case of compounds **18a**–**c** and **24** the triphenylmethane produced from the hydrogenation of trityl protecting group was completely removed by adding a small quantity of CHCl_3 _and discounting the supernatant where the polar products (**19a**–**c**, **25**) were insoluble. 

*Methyl 3-[(2S)-5-Oxopyrrolidin-2-yl] propanoate* (**7**). Prepared from alkene **6**. The product was obtained as a yellowish solid in 94% yield and high purity. Rf 0.14 in EtOAc; mp. 72–75 °C; [α]_D_ +14.3 (c 0.95, CHCl_3_). ^1^H-NMR (CDCl_3_): δ 7.01 (bs, 1H, N*H*), 3.73–3.64 (m, 1H, NC*H*), 3.66 (s, 3H, C*H*_3_), 2.41–1.60 (m, 8H, 4 × C*H*_2_). ^13^C-NMR: δ 178.6 (N*C*O), 173.1 (*C*OOCH_3_), 53.7 (N*C*H), 51.4 (*C*H_3_), 31.3, 30.1, 26.5. MS (ESI): *m/z* = 172 (100) (M+Η^+^). Anal. calcd for C_8_H_13_NO_3_: C, 56.13; H, 7.65; N, 8.18 Found: C, 56.22; H, 7.58; N, 8.22.

*1-(3-{(2R)-1-[4-(2-Methoxyethoxy)benzyl]-5-oxopyrrolidin-2-yl}propyl)-1H-imidazole-5-carboxylic acid* (**14**). Catalytic hydrogenation of compound **12** afforded product **14** as a pure colorless oil in 99% yield. Rf 0.18 in EtOAc–MeOH, 3:2. [α]_D_ +2.1 (c 1, MeOH). ^1^H-NMR (CD_3_OD): δ 8.09 (s, 1H, NC*H*N), 7.80 (s, 1H, NC*H*CCOO), 7.12 (d, *J* = 8.5 Hz, 2H, Ph), 6.88 (d, *J* = 8.5 Hz, 2H, Ph), 4.71 (d, *J* = 15.0 Hz, 1H, NCH*H*Ph), 4.41–4.34 (m, 2H, C*H*_2_NCHCOO), 4.11–3.98 (m, 3H, NCH*H*Ph, PhO*C*H_2_), 3.75–3.71 (m, 2H, C*H*_2_OCH_3_), 3.55–3.48 (m, 1H, NC*H*CH_2_), 3.41 (s, 3H, OC*H*_3_), 2.46–1.28 (m, 8H, 4 × C*H*_2_). ^13^C-NMR: δ 177.7 (N*C*O), 162.8 (COOH), 159.8, 142.0, 134.2, 130.4, 130.0, 126.5, 115.8, 72.2 (*C*H_2_OCH_3_), 68.4 (PhO*C*H_2_), 59.3 (*C*H_3_), 58.6 (N*C*H), 48.1 (*C*H_2_NCCOOH), 44.5 (N*C*H_2_Ph), 31.2, 30.5, 27.0, 24.5. MS (ESI): *m/z* = 402 (100) (M+H^+^). Anal. calcd for C_21_H_27_N_3_O_5_: C, 62.83; H, 6.78; N, 10.47 Found: C, 62.88; H, 6.73; N, 10.38.

*1-(3-{(2R)-1-[4-(2-Methoxyethoxy)benzyl]-5-oxopyrrolidin-2-yl}propyl)-1H-imidazole-4-carboxylic acid* (**15**). Catalytic hydrogenation of compound **13** afforded product **15** as a pure colorless oil in 90% yield. Rf 0.15 in EtOAc–MeOH, 1:1. [α]_D_ +0.9 (c 1, MeOH). ^1^H-NMR (CD_3_OD): δ 7.83 (s, 1H, NC*H*N), 7.68 (s, 1H, NC*H*COO), 7.10 (d, *J* = 8.2 Hz, 2H, Ph), 6.88 (d, *J* = 8.2 Hz, 2H, Ph), 4.60 (d, *J* = 14.9 Hz, 1H, NCH*H*Ph), 4.11–3.99 (m, 5H, PhO*C*H_2_, NCH*H*Ph, C*H*_2_NCHCOO), 3.75–3.71 (m, 2H, C*H*_2_OCH_3_), 3.51–3.47 (m, 1H, NC*H*), 3.41 (s, 3H, OC*H*_3_), 2.47–1.21 (m, 8H, 4 × C*H*_2_). ^13^C-NMR: δ 177.8 (N*C*O), 165.6 (COOH), 159.8, 139.5, 136.0, 134.7, 130.4, 130.0, 126.4, 115.8, 72.3 (*C*H_2_OCH_3_), 68.4 (PhO*C*H_2_), 59.3 (*C*H_3_), 58.5 (N*C*H), 48.4 (*C*H_2_NCHN), 44.7 (N*C*H_2_Ph), 31.2, 30.6, 26.8, 24.6. MS (ESI): *m/z* = 402 (100) (M+H^+^). Anal. calcd for C_21_H_27_N_3_O_5_: C, 62.83; H, 6.78; N, 10.47 Found: C, 62.74; H, 6.84; N, 10.35.

*1-[4-(2-Methoxyethoxy)benzyl]-5-oxo-L-prolyl-L-histidine* (**19a**). Prepared from compound **18a** as a colorless oil in 97% yield. Rf 0.1 in EtOAc–MeOH, 1:1. [α]_D_ +3.0 (c 1, MeOH). ^1^H-NMR (CD_3_OD): δ 8.85 (s, 1H, NC*H*N), 7.34 (s, 1H, NCHNC*H*), 7.05 (d, *J* = 8.5 Hz, 2H, Ph), 6.88 (d, *J* = 8.5 Hz, 2H, Ph), 4.84–4.72 (m, 2H, NHC*H*COO, NC*H*HPh), 4.10–4.06 (m, 2H, PhOC*H*_2_), 4.01–3.95 (m, 1H, NC*H*CON), 3.75–3.62 (m, 3H, C*H*_2_OCH_3_, NC*H*HPh), 3.42 (s, 3H, C*H*_3_), 3.15–3.02 (m, 2H, C*H_2_*CHN), 2.57–1.96 (m, 4H, 2 × C*H*_2_). ^13^C-NMR: δ 178.3 (N*C*O), 173.9, 173.2, 160.0, 135.3, 131.7, 130.8, 129.1, 118.3, 115.9, 72.2 (*C*H_2_OCH_3_), 68.5 (O*C*H_2_), 61.1 (N*C*HCON), 59.3 (O*C*H_3_), 52.7 (HN*C*HCO), 45.8 (*C*H_2_Ph), 30.8 (C*H*_2_CHN), 28.0, 24.2. MS (ESI): *m/z* = 431 (100) (M+Η^+^). Anal. calcd for C_21_H_26_N_4_O_6_: C, 58.59; H, 6.09; N, 13.02 Found: C, 58.65; H, 5.98; N, 13.14.

*1-[4-(3-Methoxypropoxy)benzyl]-5-oxo-L-prolyl-L-histidine* (**19b**). Prepared from compound **18b** as a colorless oil in 99% yield. Rf 0.11 in EtOAc–MeOH, 1:1. [α]_D_ +11.5 (c 1, MeOH). ^1^H-NMR (CD_3_OD): δ 8.75 (s, 1H, NC*H*N), 7.32 (s, 1H, NC*H*NC), 7.04 (d, *J* = 8.5 Hz, 2H, Ph), 6.85 (d, *J* = 8.5 Hz, 2H, Ph), 4.88 (d, *J* = 14.8 Hz, 1H, NCH*H*Ph), 4.75–4.65 (m, 1H, NHC*H*COO), 4.04–3.98 (m, 1H, NC*H*CO), 4.01 (t, *J* = 6.3 Hz, 2H, PhOC*H*_2_), 3.63 (d, *J* = 14.8 Hz, 1H, NCH*H*Ph), 3.54 (t, *J* = 6.2 Hz, 2H, C*H*_2_OCH_3_), 3.33 (s, 3H, OC*H*_3_), 3.28–3.03 (m, 2H, C*H_2_*CN), 2.53–1.93 (m, 6H, 3 × C*H*_2_). ^13^C-NMR: δ 178.3 (N*C*O), 173.7, 160.2, 135.2, 132.1, 131.0, 130.7, 128.9, 118.2, 115.8, 70.3 (*C*H_2_OCH_3_), 65.9 (PhO*C*H_2_), 61.2 (N*C*H), 58.9 (O*C*H_3_), 53.4 (*C*HCOOH), 45.8 (N*C*H_2_Ph), 30.9, 30.6, 28.4, 24.2. MS (ESI): *m/z* = 445 (100) (M+Η^+^). Anal. calcd for C_22_H_28_N_4_O_6_: C, 59.45; H, 6.35; N, 12.61 Found: C, 59.52; H, 6.37; N, 12.50.

*1-[4-(4-Methoxybutoxy)benzyl]-5-oxo-L-prolyl-L-histidine* (**19c**). Prepared from compound **18c** as a colorless oil in 92% yield. Rf 0.16 in EtOAc–MeOH, 1:1. [α]_D_ +6.9 (c 1, MeOH). ^1^H-NMR (CD_3_OD): δ 8.84 (s, 1H, HNC*H*N), 7.36 (s, 1H, NCC*H*N), 7.04 (d, *J* = 8.5 Hz, 2H, Ph), 6.85 (d, *J* = 8.5 Hz, 2H, Ph), 4.89 (d, *J* = 14.7 Hz, 1H, NCH*H*Ph), 4.80–4.73 (m, 1H, NHC*H*COO), 4.02–3.96 (m, 1H, NC*H*), 3.95 (t, *J* = 5.4 Hz, 2H, PhOC*H*_2_), 3.63 (d, *J* = 14.7 Hz, 1H, NCH*H*Ph), 3.45 (t, *J* = 6.1 Hz, 2H, C*H*_2_OCH_3_), 3.33 (s, 3H, C*H*_3_), 3.17–3.05 (m, 2H, C*H_2_*CNH), 2.49-1.96 (m, 4H, 2 × C*H*_2_), 1.84–1.65 (m, 4H, (OCH_2_C*H*_2_)_2_). ^13^C-NMR: δ 178.2 (N*C*O), 173.9, 160.3, 135.2, 131.8, 131.1, 130.7, 128.8, 118.3, 115.8, 73.5 (*C*H_2_OCH_3_), 68.9 (PhO*C*H_2_), 61.1 (N*C*H), 58.8 (O*C*H_3_), 52.9 (*C*HCOOH), 45.8 (N*C*H_2_Ph), 30.8, 28.0, 27.3, 27.2, 24.1. MS (ESI): *m/z* = 459 (100) (M+Η^+^). Anal. calcd for C_23_H_30_N_4_O_6_: C, 60.25; H, 6.59; N, 12.12 Found: C, 60.32; H, 6.47; N, 12.30.

*Methyl 1-(4-Hydroxybenzyl)-5-oxo-L-prolinate* (**21**). Prepared from compound **20** as a colorless oil in 99% yield. Rf 0.69 in EtOAc. [α]_D_ +35.5 (c 1.1, CHCl_3_). ^1^H-NMR (CDCl_3_): δ 7.03 (d, *J* = 7.5 Hz, 2H, Ph), 6.80 (d, *J* = 7.5 Hz, 2H, Ph), 4.89 (d, *J* = 14.5 Hz, 1H, NCH*H*Ph), 4.02–3.91 (m, 2H, NCH*H*Ph, NC*H*), 3.67 (s, 3H, C*H*_3_), 2.55–2.04 (m, 4H, 2xC*H*_2_). ^13^C-NMR: δ 175.7 (N*C*O), 172.2 (*C*OOCH_3_), 156.3, 130.0, 126.5, 115.7, 58.9 (N*C*H), 52.5 (COO*C*H_3_), 45.3 (N*C*HPh), 29.8, 22.7. MS (ESI): *m/z* = 250 (97) (M+Η^+^). Anal. calcd for C_13_H_15_NO_4_: C, 62.64; H, 6.07; N, 5.62 Found: C, 62.59; H, 6.17; N, 5.54.

*1-(4-{[(4R)-3-(tert-Butoxycarbonyl)-2,2-dimethyl-1,3-oxazolidin-4-yl]methoxy}benzyl)-5-oxo-L-prolyl-L-histidine* (**25**). Prepared from compound **24** as a colorless oil in 97% yield. Rf 0.18 in EtOAc–MeOH, 1:1. [α]_D_ −24.9 (c 0.8, MeOH). ^1^H-NMR (CD_3_OD): δ 8.90 (s, 1H, HNC*H*N), 7.40 (s, 1H, HNCHNC*H*), 7.05 (d, *J* = 8.3 Hz, 2H, Ph), 6.93 (d, *J* = 8.3 Hz, 2H, Ph), 4.87–4.75 (m, 2H, C*H*COOH, NCH*H*Ph), 4.17–3,81 (m, 6H, NC*H*CO, PhOC*H*_2_C*H*C*H*_2_O), 3.64 (d, *J* = 15.0 Hz, 1H, NCH*H*Ph), 3.41–3.06 (m, 2H,HOOCCHC*H*_2_), 2.58–1.94 (m, 4H, 2xC*H*_2_), 1.58–1.45 (m, 15H, 5 × C*H*_3_). ^13^C-NMR: δ 173.9 (N*C*O), 172.8, 159.8, 153.4 (N*C*OO), 135.3, 131.5, 130.8, 129.5, 129.3, 118.4, 115.9, 95.0 (N*C*(CH_3_)_2_O), 82.2 (*C*(CH_3_)_3_), 67.3, 66.2, 61.1 (N*C*HCO), 57.5 (PhO*C*H_2_), 52.5 (NH*C*HCOOH), 45.8 (N*C*H_2_Ph), 30.8, 28.8 (*C*H_3_), 27.9, 27.7, 24.5, 24.2. MS (ESI): *m/z* = 586 (100) (M+Η^+^). Anal. calcd for C_29_H_39_N_5_O_8_: C, 59.47; H, 6.71; N, 11.96 Found: C, 59.52; H, 6.68; N, 12.05.

### 3.7. General Procedure for the Preparation of Compounds ***17a**–**c**, **23***

To a stirred solution of methylester (compounds **16a**–**c**, **22**) (1 mmol), in MeOH (3 mL), an aq. solution of 2N NaOH (0.5 ml, 1 mmol) was added and the reaction mixture was left stirring at rt for 2–3 h. Upon completion of the reaction, the MeOH was evaporated under reduced pressure and the residual product was diluted with H_2_O and extracted with Et_2_O (1 × 10 mL). The aqueous phase was acidified with 1 N HCl aq. solution at pH 2 and extracted with EtOAc (2 × 10 mL). The combined organic phases were neutralized by washing with brine and H_2_O, dried over Na_2_SO_4_ and evaporated under reduced pressure to afford the carboxylic products in quantitative yield and high purity.

*1-[4-(2-Methoxyethoxy)benzyl]-5-oxo-L-proline* (**17a**). Prepared from methylester **16a** as a colorless oil in 94% yield. Rf 0.36 in EtOAc–MeOH, 3:2. [α]_D_ +43.8 (c 1, CHCl_3_). ^1^H-NMR (CDCl_3_): δ 8.77 (bs, 1H, COO*H*), 7.13 (d, *J* = 8.6 Hz, 2H, Ph), 6.85 (d, *J* = 8.6 Hz, 2H, Ph), 5.02 (d, *J* = 14.7 Hz, 1H, CH*H*Ph), 4.11–4.06 (m, 2H, PhOC*H*_2_), 3.99–3.93 (m, 1H, NC*H*COO), 3.91 (d, *J* = 4.7 Hz, 1H, CH*H*Ph), 3.77–3.72 (m, 2H, C*H*_2_OCH_3_), 3.44 (s, 3H, OC*H*_3_), 2.68–2.09 (m, 4H, 2 × C*H*_2_). ^13^C-NMR: δ 176.2 (N*C*O), 174.1(*C*OOH), 158.4, 129.9, 127.6, 114.7, 70.9 (*C*H_2_OCH_3_), 67.1 (O*C*H_2_), 59.1 (N*C*H), 58.5 (O*C*H_3_), 45.0 (*C*H_2_Ph), 29.6, 22.8. MS (ESI): *m/z* = 292 (100) (M−Η^+^). Anal. calcd for C_15_H_19_NO_5_: C, 61.42; H, 6.53; N, 4.78 Found: C, 61.38; H, 6.57; N, 4.82.

*1-[4-(3-Methoxypropoxy)benzyl]-5-oxo-L-proline* (**17b**). Prepared from methylester **16b**, as a colorless oil in 99% yield. Rf 0.24 in EtOAc–MeOH, 3:2. [α]_D_ +17.2 (c 1, CHCl_3_). ^1^H-NMR (CDCl_3_): δ 9.62 (bs, 1H, COO*H*), 7.13 (d, *J* = 8.6 Hz, 2H, Ph), 6.83 (d, *J* = 8.6 Hz, 2H, Ph), 5.05 (d, *J* = 4.7 Hz, 1H, CH*H*Ph), 4.04–3.94 (m, 3H, NC*H*COO, PhOC*H*_2_), 3.91 (d, *J* = 14.7 Hz, 1H, CH*H*Ph), 3.56 (t, *J* = 6.2 Hz, 2H, C*H*_2_OCH_3_), 3.35 (s, 3H, OC*H*_3_), 2.66–2.09 (m, 4H, 2 × C*H*_2_), 2.09–1.96 (m, 2H, CH_2_C*H*_2_CH_2_). ^13^C NMR: δ 176.2 (N*C*O), 174.2 (COO*H*), 158.6, 129.9, 127.2, 114.6, 69.2 (*C*H_2_OCH_3_), 64.7 (PhO*C*H_2_), 58.6 (N*C*H), 58.5 (O*C*H_3_), 45.0 (*C*H_2_Ph), 29.7, 29.4, 22.8. MS (ESI): *m/z* = 306 (100) (M−Η^+^). Anal. calcd for C_16_H_21_NO_5_: C, 62.53; H, 6.89; N, 4.56 Found: C, 62.58; H, 6.77; N, 4.53.

*1-[4-(4-Methoxybutoxy)benzyl]-5-oxo-L-proline* (**17c**). Prepared from methylester **16c**, as a colorless oil in 96% yield. Rf 0.30 in EtOAc–MeOH, 3:2. [α]_D_ +13.3 (c 1, CHCl_3_). ^1^H-NMR (CDCl_3_): δ 9.98 (bs, 1H, COO*H*), 7.12 (d, *J* = 8.6 Hz, 2H, Ph), 6.81 (d, *J* = 8.6 Hz, 2H, Ph), 5.03 (d, *J* = 14.7 Hz, 1H, NCH*H*Ph), 4.00–3.88 (m, 4H, NC*H*HPh, NC*H*, PhOC*H*_2_), 3.44 (t, *J* = 6.2Hz, 2H, C*H*_2_OCH_3_), 3.34 (s, 3H, OC*H*_3_), 2.65–2.08 (m, 4H, 2xC*H*_2_), 1.85–1.68 (m, 4H, (OC*H*_2_CH_2_)_2_). ^13^C-NMR: δ 176.2 (N*C*O), 173.8 (*C*OO), 158.6, 129.9, 127.1, 114.6, 72.3 (*C*H_2_OCH_3_), 67.5 (PhO*C*H_2_), 58.4 (N*C*H), 58.3 (O*C*H_3_), 45.0 (*C*H_2_Ph), 29.6, 26.0, 25.8, 22.8. MS (ESI): *m/z* = 320 (100) (M−Η^+^). Anal. calcd for C_17_H_23_NO_5_: C, 63.54; H, 7.21; N, 4.36 Found: C, 63.38; H, 7.19; N, 4.42.

*1-(4-{[(4R)-3-(tert-Butoxycarbonyl)-2,2-dimethyl-1,3-oxazolidin-4-yl]methoxy}benzyl)-5-oxo-L-proline* (**23**). Prepared from methylester **22**, as a colorless oil in 94% yield. Rf 0.43 in EtOAc–MeOH, 1:1. [α]_D_ −11.5 (c 1, CHCl_3_). ^1^H-NMR (CDCl_3_): δ 7.62 (bs, 1H, COO*H*), 7.13 (d, *J* = 7.4 Hz, 2H, Ph), 6.88 (d, *J* = 4 Hz, 2H, Ph), 5.03 (dd, *J_1 _*= 14.6 Hz, *J_2_* = 3.1 Hz, 1H, NCH*H*Ph), 4.28–3.80 (m, 7H, NCH*H*Ph, NC*H*COOH, OC*H*_2_C*H*C*H*_2_O), 4.68–2.15 (m, 4H, 2 × C*H*_2_), 1.64-1.52 (m, 15H, 5 × C*H*_3_). ^13^C-NMR: δ 176.1 (N*C*O), 173.9 (*C*OOH), 158.1, 152.4 (N*C*OO), 129.9, 127.6, 114.8, 93.6 (O*C*(CH_3_)_2_N), 80.9 (*C*(CH_3_)_3_), 66.0, 65.1, 58.4 (N*C*HCOOH), 55.9 (PhO*C*H_2_), 45.0 (N*C*H_2_Ph), 29.6, 28.3 (*C*H_3_), 27.4, 24.2, 22.8. MS (ESI): *m/z* = 447 (100) (M-Η^+^). Anal. calcd for C_23_H_32_N_2_O_7_: C, 61.59; H, 7.19; N, 6.25 Found: C, 61.53; H, 7.24; N, 6.32.

### 3.8. General Procedure for the Preparation of Compounds ***18a**–**c**, **24***

To a stirred solution of carboxylic acids (compounds **17a**–**c**, **23**) (1 mmol) in CH_2_Cl_2_ (10 mL) at 0 °C, 1-hydroxybenzotriazole (HOBt) (0.135 g, 1 mmol), freshly prepared benzyl 3-trityl-L-histidinate (**42**) (0.537 g, 1.1 mmol), Et_3_N (0.154 mL, 1.1 mmol) and *N*-ethyl-*N*-dimethylaminopropyl-carbodiimide hydrochloride (EDC.HCl) (0.210 g, 1.1 mmol) were added consecutively. The reaction mixture was left stirring at 0 °C for 1 h and then warmed to room temperature and left stirring for 18 h. The solvents were evaporated under reduced pressure and the crude product was dissolved in EtOAc (20 mL). The organic layer was washed with 5% aq. H_2_SO_4_, H_2_O, 5% aq. NaHCO_3_, and brine. After drying over Na_2_SO_4_ and evaporation of the solvent *in vacuo*, the crude ester was purified using column chromatography with the appropriate solvent systems as it will be defined in each case below.

*Benzyl 1-[4-(2-Methoxyethoxy)benzyl]-5-oxo-L-prolyl-3-trityl-L-histidinate* (**18a**). Prepared from compound **17a**. Eluent EtOAc–MeOH 9:1. Yield: 55% (colorless oil); Rf 0.55 in EtOAc–MeOH 9:1; [α]_D_ +3.8 (c 1, CHCl_3_). ^1^H-NMR (CDCl_3_): δ 8.22 (d, *J* = 7.8 Hz, 1H, N*H*), 7.39 (d, *J* = 1.3 Hz, 1H, NC*H*N), 7.33–7.03 (m, 22H, Ph), 6.80 (d, *J* = 8.6 Hz, 2H, Ph), 6.47 (s, 1H, NCHNC*H*C), 5.01 (d, *J* = 14.3 Hz, 1H, CH*H*Ph), 5.00 (s, 2H, OC*H_2_*Ph), 4.86–4.77 (m, 1H, NHC*H*COO), 4.12–4.04 (m, 2H, PhOC*H*_2_), 3.89–3.83 (m, 1H, NC*H*CON), 3.75 (d, *J* = 14.3 Hz, 1H, CH*H*Ph), 3.74–3.69 (m, 2H, C*H*_2_OCH_3_), 3.43 (s, 3H, OC*H*_3_), 3.13–2.91 (m, 2H, COCHC*H_2_*CHN), 2.68–1.98 (m, 4H, 2 × C*H*_2_). ^13^C-NMR: δ 175.5 (N*C*O), 171.6, 170.6, 158.2, 142.0, 138.7, 135.9, 135.2, 129.9, 129.6, 128.5, 128.4, 128.3, 128.2, 128.1, 119.5, 119.4, 114.6, 75.4 (N*C*Ph), 70.9 (*C*H_2_OCH_3_), 67.1 (O*C*H_2_), 67.0 (O*C*H_2_Ph), 60.0 (N*C*H), 59.2 (O*C*H_3_), 52.5 (HN*C*HCO), 44.5 (*C*H_2_Ph), 29.7, 28.8 (NHCHC*H*_2_C), 23.2. MS (ESI): *m/z* = 763 (100) (M+Η^+^). Anal. calcd for C_47_H_46_N_4_O_6_: C, 74.00; H, 6.08; N, 7.34 Found: C, 74.15; H, 6.16; N, 7.27.

*Benzyl 1-[4-(3-Methoxypropoxy)benzyl]-5-oxo-L-prolyl-3-trityl-L-histidinate* (**18b**). Prepared from compound **17b**. Eluent EtOAc–MeOH 9:1. Yield: 68% (colorless oil); Rf 0.63 in EtOAc–MeOH 9:1; [α]_D_ +8.5 (c 1, CHCl_3_). ^1^H-NMR (CDCl_3_): δ 8.19 (d, *J* = 7.8 Hz, 1H, N*H*), 7.40 (d, *J* = 1.3 Hz, 1H, NC*H*N), 7.34–7.04 (m, 22H, Ph), 6.78 (d, *J* = 8.7 Hz, 2H, Ph), 6.48 (d, *J* = 1.2 Hz, 1H, NC*H*CN), 5.02 (d, *J* = 14.5 Hz, 1H, NCH*H*Ph), 5.01 (s, 2H, C*H_2_*Ph), 4.00 (t, *J* = 6.3 Hz, 2H, PhOC*H*_2_), 3.89–3.83 (m, 1H, NC*H*CO), 3.76 (d, *J* = 14.5 Hz, 1H, NCH*H*Ph), 3.53 (t, *J* = 6.2 Hz, 2H, CH_2_OC*H*_3_), 3.34 (s, 3H, OC*H*_3_), 3.14–2.92 (m, 2H, HNCHCH_2_), 2.69–2.08 (m, 4H, 2 × C*H*_2_), 2.05–1.95 (m, 2H, CH_2_C*H*_2_CH_2_). ^13^C-NMR: δ 175.5 (N*C*O), 171.6, 170.6, 158.4, 142.1, 138.7, 136.0, 135.2, 130.0, 129.6, 128.5, 128.3, 128.2, 128.1, 128.0, 119.5, 114.5, 75.4 (*C*Ph), 69.2 (*C*H_2_OCH_3_), 67.0 (O*C*H_2_Ph), 64.7 (PhO*C*H_2_), 60.0 (N*C*H), 58.7 (O*C*H_3_), 52.5 (HN*C*HCO), 44.5 (*C*H_2_Ph), 29.8, 29.5 (CH_2_C*H*_2_CH_2_), 28.9 (NHCHC*H*_2_C), 23.2. MS (ESI): *m/z* = 777 (100) (M+Η^+^). Anal. calcd for C_48_H_48_N_4_O_6_: C, 74.21; H, 6.23; N, 7.21 Found: C, 74.11; H, 6.32; N, 7.28.

*Benzyl 1-[4-(4-Methoxybutoxy)benzyl]-5-oxo-L-prolyl-3-trityl-L-histidinate* (**18c**). Prepared from compound **17c**. Eluent EtOAc–MeOH 9:1. Yield: 63% (colorless oil); Rf 0.56 in EtOAc–MeOH 9:1; [α]_D_ +3.8 (c 1, CHCl_3_). ^1^H-NMR (CDCl_3_): δ 8.21 (d, *J* = 7.9 Hz, 1H, N*H*), 7.40 (d, *J* = 1.3 Hz, 1H, NC*H*N), 7.35–7.04 (m, 22H, Ph), 6.76 (d, *J* = 8.6 Hz, 2H, Ph), 6.48 (d, *J* = 1.1 Hz, 1H, NC*H*CN), 5.02 (d, *J* = 14.5 Hz, 1H, NCH*H*Ph), 5.01 (s, 2H, COOC*H_2_*Ph), 4.87–4.79 (m, 1H, NC*H*COO), 3.92 (t, *J* = 5.9 Hz, 2H, PhOC*H*_2_), 3.88–3.83 (m, 1H, NC*H*), 3.76 (d, *J* = 14.5 Hz, 1H, NCH*H*Ph), 3.43 (t, *J* = 6.1 Hz, 2H, CH_2_OC*H*_3_), 3.34 (s, 3H, OC*H*_3_), 3.13–2.92 (m, 2H, CHC*H*_2_CN), 2.65–1.98 (m, 4H, 2 × C*H*_2_), 1.86–1.65 (m, 4H, (OCH_2_C*H*_2_)_2_). ^13^C-NMR: δ 175.4 (N*C*O), 171.6, 170.5, 158.4, 142.0, 138.7, 135.9, 135.2, 129.9, 129.6, 128.4, 128.3, 128.1, 128.0, 127.9, 119.4, 114.5, 75.3 (*C*Ph), 72.2 (*C*H_2_OCH_3_), 67.5 (O*C*H_2_Ph), 66.9 (PhO*C*H_2_), 59.9 (N*C*H), 58.5 (O*C*H_3_), 52.5 (HN*C*HCO), 44.5 (*C*H_2_Ph), 29.7, 28.8, 26.1, 25.9, 23.2. MS (ESI): *m/z* = 791 (100) (M+Η^+^). Anal. calcd for C_49_H_50_N_4_O_6_: C, 74.41; H, 6.37; N, 7.08 Found: C, 74.35; H, 6.42; N, 7.18.

*Benzyl 1-(4-{[(4R)-3-(tert-Butoxycarbonyl)-2,2-dimethyl-1,3-oxazolidin-4-yl]methoxy}benzyl)-5-oxo-L-prolyl-3-trityl-L-histidinate* (**24**). Prepared from compound **23**. Eluent EtOAc-petroleum ether (bp. 40–60 °C) 9:1, followed by EtOAc. Yield: 60% (colorless oil); Rf 0.64 in EtOAc; [α]_D_ −16.4 (c 1, CHCl_3_). ^1^H-NMR (CDCl_3_): δ 8.23 (t, *J* = 8.3 Hz, 1H, N*H*), 7.40–6.80 (m, 25H, Ph), 6.47 (s, 1H, Ph), 5.05–4.98 (m, 3H, COOC*H*_2_, NCH*H*Ph), 4.86–4.77 (m, 1H, HNC*H*COO), 4.26–3.69 (m, 7H, NCH*H*Ph, NC*H*CONH, PhOC*H*_2_C*H*C*H*_2_O), 3.13–2.92 (m, 2H, HNCHC*H*_2_C), 2.68–2.03 (m, 4H, 2×C*H*_2_), 1.61–1.48 (m, 15H, 5 × C*H*_3_). ^13^C-NMR: δ 175.5 (N*C*O), 171.6, 170.5, 158.0, 152.2 (N*C*OO), 142.0, 138.6, 135.8, 135.2, 130.0, 129.9, 129.6, 128.6, 128.5, 128.3, 128.1, 119.6, 114.7, 93.5 (O*C*(CH_3_)_2_N), 80.2 (*C*(CH_3_)_3_), 75.4 (*C*Ph_3_), 67.0 (COO*C*H_2_Ph), 66.1, 65.3, 60.0 (N*C*HCO), 55.7 (PhO*C*H_2_), 52.5 (NH*C*HCOO), 44.5 (N*C*H_2_Ph), 29.7, 28.5 (*C*H_3_), 27.5, 24.2, 23.3, 23.0. MS (ESI): *m/z* = 919 (100) (M+Η^+^). Anal. calcd for C_55_H_59_N_5_O_8_: C, 71.95; H, 6.48; N, 7.63 Found: C, 72.07; H, 6.24; N, 7.58.

*Μethyl 1-(4-{[(4R)-3-(tert-Butoxycarbonyl)-2,2-dimethyl-1,3-oxazolidin-4-yl]methoxy}benzyl)-5-oxo-L-prolinate *(**22**). To a stirred solution of alcohol (*tert*-butyl (4*R*)-4-(hydroxymethyl)-2,2-dimethyl-1,3-oxazolidine-3-carboxylate) [33], (0.231g, 1 mmol) in dry toluene (5 mL), compound **21** (0.249 g, 1 mmol), DEAD (0.19 mL, 1.2 mmol), and PPh_3_ (0.314 g, 1.2 mmol) were added consecutively and the reaction mixture was refluxed for 15 h under argon. After evaporation of the solvent under reduced pressure the product was purified using column chromatography and EtOAc–petroleum ether (bp. 40–60 °C) 7:3 as eluent and isolated as a colorless oil in 60% yield (0.278 g). Rf 0.45 in EtOAc–petroleum ether (bp. 40–60 °C) 7:3; [α]_D_ −17.7 (c 1, CHCl_3_). ^1^H-NMR (CDCl_3_): δ 7.10 (d, *J* = 7.9 Hz, 2H, Ph), 6.70 (bs, 2H, Ph), 4.94 (d, *J* = 14.6 Hz, 1H, NCH*H*Ph), 4.21–3.81 (m, 7H, NCH*H*Ph, NC*H*COOCH_3_, PhOC*H*_2_C*H*C*H*_2_O), 3.67 (s, 3H, OC*H*_3_), 2.64–2.02 (m, 4H, 2 × C*H*_2_), 1.60–1.48 (m, 15H, 5 × C*H*_3_). ^13^C-NMR: δ 174.8 (N*C*O), 172.2 (*C*OOCH_3_), 158.1, 152.2 (N*C*OO), 129.8, 127.9, 114.7, 93.4 (N*C*(CH_3_)_2_O), 80.4 (*C*(CH_3_)_3_), 66.0, 65.1, 58.5 (N*C*HCOO), 55.6 (PhO*C*H_2_), 52.3 (COO*C*H_3_), 44.8 (N*C*HPh), 29.5, 28.3 (C(*C*H_3_)_3_), 27.4, 24.2, 22.6. MS (ESI): *m/z* = 480 (100) (M+NΗ_4_^+^). Anal. calcd for C_24_H_34_N_2_O_7_: C, 62.32; H, 7.41; N, 6.06 Found: C, 62.28; H, 7.39; N, 6.15.

*1-(4-{[(2S)-2-Ammonio-3-Hydroxypropyl]oxy}benzyl)-5-oxo-L-prolyl-L-histidine hydrochloride* (**26**). To a stirred solution of compound **25** (21 mg, 0.036 mmol) in dioxane (1 mL) at 0 °C, a 4 M solution of HCl in dioxane (0.108 mL, 0.432 mmol) was added and the reaction mixture was left stirring at room temperature for 1 h. After evaporation of the solvent under reduced pressure, the product was precipitated by the addition of Et_2_O and recrystallized from dioxane/Et_2_O twice. It was obtained as a white gummy solid in 90% yield (16 mg). [α]_D_ +3.4 (c 0.7, H_2_O). ^1^H-NMR (D_2_O): δ 8.87 (s, 1H, HNC*H*N), 7.32 (s, 1H, HNCHNC*H*), 7.14 (d, *J* = 8.4 Hz, 2H, Ph), 6.99 (d, *J* = 8.4 Hz, 2H, Ph), 4.75–4.70 (m, 2H, C*H*COOH, NCH*H*Ph), 4.33–4.19 (m, 2H, PhOC*H*_2_), 4.16–4.12 (m, 1H, NC*H*), 3.98–3.80 (m, 4H, NCH*H*Ph, C*H*NH_2_, C*H*_2_OH), 3.33 (dd, *J_1_* = 5.4 Hz, *J_2 _*= 15.6 Hz, 1H, HNCHCH*H*C), 3.15 (dd, *J_1_* = 9.0 Hz, *J_2_* = 15.5 Hz, 1H, HNCHCH*H*C), 2.61-1.87 (m, 4H, 2 × C*H*_2_). ^13^C-NMR: δ 179.6 (N*C*O), 174.2, 173.6, 158.0, 134.2, 130.3, 129.5, 128.7, 117.7, 115.5, 65.6 (PhO*C*H_2_), 61.3 (N*C*HCO), 59.3 (*C*H_2_OH), 52.8, 52.3, 45.7 (N*C*H_2_Ph), 30.4, 26.6, 23.2. MS (ESI): *m/z* = 446 (100) (M+Η^+^). Anal. calcd for C_21_H_28_N_5_O_6_Cl: C, 52.34; H, 5.86; N, 14.53 Found: C, 52.28; H, 5.92; N, 14.47.

*Benzyl N-[(9H-Fluoren-9-ylmethoxy)carbonyl]-1-trityl-L-histidinate* (**41**). To a solution of Fmoc-*S*-His(Trt)-OH (0.20 g, 0.33 mmol) in DMF (1 mL) Cs_2_CO_3_ (53 mg, 0.162 mmol) was added with some drops of H_2_O to dissolve the inorganic salt. The solvent was evaporated under reduced pressure to dryness and the residue, dissolved in DMF (3 mL) was stirred for 5 min at rt, followed by the addition of benzyl bromide (44 μL, 0.371 mmol). After stirring overnight at rt and evaporation of DMF under reduced pressure, the residue was dissolved in EtOAc and the organic phase was washed with H_2_O, 5% aq. solution of NaHCO_3_ and H_2_O. The organic layer was dried over Na_2_SO_4_, evaporated in vacuo and the residue was purified by column chromatography on silica gel, eluting with EtOAc-petroleum ether (bp. 40–60 °C) 1:1. The product was isolated as colorless oil in 87% yield (0.20 g). Rf 0.50 in EtOAc–petroleum ether (bp. 40–60 °C) 1:1; [α]_D_ −2.7 (c 1, CHCl_3_). ^1^H-NMR (CDCl_3_): δ 7.77–7.06 (m, 29H, Ph, NC*H*N), 6.61 (d, *J* = 8.2 Hz, 1H, N*H*), 6.54 (s, 1H, NCHNC*H*C), 5.07 (dd, *J_1 _*= 12.5 Hz, *J_2 _*= 14.9 Hz, 2H, OC*H_2_*Ph), 4.73–4.64 (m, 1H, HNC*H*CO), 4.40–4.19 (m, 3H, C*H*C*H*_2_OCO), 3.12 (d, *J* = 5.0Hz, 2H, C*H_2_*CHN). ^13^C-NMR: δ 171.2 (HNCH*C*OO), 156.2 (*C*OONH), 144.1, 143.9, 142.0, 141.2, 138.4, 135.8, 135.4, 129.7, 128.5, 128.2, 128.1, 127.6, 127.0, 125.4, 125.3, 119.8, 75.6 (*C*Ph), 67.2 (*C*H_2_COONH), 66.9 (O*C*H_2_Ph), 54.3 (HN*C*HCOO), 47.1 (C*H*CH_2_COO), 29.7 (*C*H_2_CHN). MS (ESI): *m/z* = 710 (100) (M+Η^+^). Anal. calcd for C_47_H_39_N_3_O_4_: C, 79.53; H, 5.54; N, 5.92 Found: C, 79.60; H, 5.58; N, 5.85.

*Benzyl-3-trityl-L-histidinate* (**42**). Compound **41** (0.15 g, 0.21 mmol) was dissolved in EtOH (1.5 mL) and Et_2_NH (153 μL, 1.48 mmol) was added. After leaving overnight at rt the solvent was evaporated under reduced pressure. The crude product was redissolved in water, washed by EtOAc (3 × 5mL) and the combined organic layers were dried over Na_2_SO_4_ and evaporated in vacuo. After purification by column chromatography and using EtOAc–MeOH (9:1) as eluent the product was isolated as colorless oil in 82% yield (84 mg). Rf 0.37 in EtOAc–MeOH 9:1; [α]_D_ −11.7 (c 1, CHCl_3_). ^1^H-NMR (CDCl_3_): δ 7.32–7.08 (m, 21H, 4 × Ph, NC*H*N), 6.54 (s, 1H, NCHNC*H*C), 5.04 (dd, *J_1_* = 12.3 Hz, *J_2_* = 20.8 Hz, 2H, OC*H_2_*Ph), 3.90–3.85 (m, 1H, H_2_NC*H*), 3.08–2.82 (m, 2H, C*H_2_*CHN). ^13^C-NMR: δ 174.4 (*C*OO), 142.3, 138.6, 137.0, 135.6, 129.7, 128.5, 128.3, 128.2, 128.1, 128.0, 119.5, 75.2 (*C*Ph), 66.6 (*C*H_2_Ph), 54.6 (H_2_N*C*H), 32.9 (*C*H_2_CHN). MS (ESI): *m/z* = 488 (100) (M+Η^+^). Anal. calcd for C_32_H_29_N_3_O_2_: C, 78.82; H, 5.99; N, 8.62 Found: C, 78.68; H, 5.79; N, 8.74.

## 4. Conclusions

In this paper the synthesis of new, optically active 2-pyrrolidinones starting from the natural chiral synthon of *S*-pyroglutamic acid is described. 

## Figures and Tables

*Reagents and Conditions*: (**i**) NaBH_4_, dry EtOH; 94%; (**ii**) TES-Cl, Et_3_N, DMAP in CH_2_Cl_2_; 83%; (**iii**) NaH, Z-Cl in dry THF; 50%; (**iv**) TFA in CH_2_Cl_2_; 86%; (**v**) Moffatt oxidation: EDC.HCl, Pyr, TFA drops in dry Tol and dry DMSO, under Ar; (**vi**) Ph_3_P=CHCOOMe in dry THF, 1h reflux under Ar; 60%; (**vii**) H_2_, 10% Pd/C in MeOH, overnight; 97%; (**viii**) **30**, NaH in dry DMF; 40%; (**ix**) LiBH_4_, dry THF; 87%; (**x**) TosCl, Et_3_N in CH_2_Cl_2_; 72%; (**xi**) , Cs_2_CO_3_ in dry DMF; 58%; (**xii**)  Cs_2_CO_3_ in dry DMF; 55% for **12**; 30% for **13**; (**xiii**) H_2_, 10% Pd/C in MeOH; 99% for **14**; 90% for **15**.

*Reagents and Conditions*: (**i**) **30**, **35**, **39**, NaH in dry DMF; 66–73%; (**ii**) 2N NaOH in MeOH; 94–99%; (**iii**) **42**, Et_3_N, HOBt, EDC.HCl in CH_2_Cl_2_; 55–68%; (**iv**) H_2_, 10% Pd/C; 92–99%.

*Reagents and Conditions*: (**i**) , NaH in dry THF; 47%; (**ii**) H_2_, 10% Pd/C in MeOH; 99%; (**iii**) , PPh_3_, DEAD in dry Tol; 60%; (**iv**) 2N NaOH in MeOH; 94%;(**v**) **42**, Et_3_N, HOBt, EDC.HCl in CH_2_Cl_2_; 48%; (**vi**) H_2_, 10% Pd/C in MeOH; 97%; (**vii**) 4M HCl/dioxane; 90%.

*Reagents and Conditions*: (**i**) PBr_3_ in Et_2_O; 58%; (**ii**) , K_2_CO_3_, 18-Crown-6 in acetone; 76%; (**iii**) PBr_3_ in Et_2_O; 60%.

*Reagents and Conditions*: (**i**) , K_2_CO_3_ in acetone; 40%; (**ii**) 1N MeONa/MeOH; 38%; (**iii**) NaBH_4_ in dry THF; 80%; (**iv**) PBr_3_ in Et_2_O; 70%.

*Reagents and Conditions*: (**i**) PBr_3_ in Et_2_O; 60%; (**ii**)  K_2_CO_3_, 18-Crown-6 in acetone; 67%; (iii) TMS-Cl, NaBr in CH_3_CN; 42%.

*Reagents and Conditions*: (**i**) Bn-Br, Cs_2_CO_3_ in DMF; 87%; (**ii**) Et_2_NH in abs. EtOH, 82%.
